# Genotypic and Phenotypic Analysis in Chinese Cohort With Autosomal Recessive Osteogenesis Imperfecta

**DOI:** 10.3389/fgene.2020.00984

**Published:** 2020-09-15

**Authors:** Shan Li, Yixuan Cao, Han Wang, Lulu Li, Xiuzhi Ren, Huan Mi, Yanzhou Wang, Yun Guan, Feiyue Zhao, Bin Mao, Tao Yang, Yi You, Xin Guan, Yujiao Yang, Xue Zhang, Xiuli Zhao

**Affiliations:** ^1^Department of Medical Genetics, Institute of Basic Medical Sciences, Chinese Academy of Medical Sciences and School of Basic Medicine, Peking Union Medical College, Beijing, China; ^2^The People’s Hospital of Wuqing District, Tianjin, China; ^3^Shandong Provincial Hospital Affiliated to Shandong University, Jinan, China; ^4^Department of Anesthesiology and Critical Care Medicine, Johns Hopkins University School of Medicine, Baltimore, MD, United States

**Keywords:** autosomal recessive osteogenesis imperfecta, mutation spectrum, phenotype, *WNT1*, Chinese cohort

## Abstract

Osteogenesis imperfecta (OI) is a rare heritable skeletal disorder which is mainly caused by defected type I collagen. Autosomal recessive OI (AR-OI) is caused by mutations of genes that are responsible for type I collagen modification and folding, and is often associated with more severe phenotypes. Due to the limited number of recessive OI patients, it has been difficult to study the mutation spectrum as well as the correlation of genotype and phenotype. This study recruited a Chinese cohort of 74 AR-OI families, aiming to establish the mutation spectrum and to examine the genotypic and phenotypic correlation. We identified 82 variants including 25 novel variants and 57 HGMD reported variants in these AR-OI patients, using whole exome sequencing/panel sequencing combined with Sanger sequencing. Pathogenic mutations were found at *WNT1* (*n* = 30, 40.54%), *SERPINF1* (*n* = 22, 29.73%), *FKBP10* (*n* = 10, 13.51%), *CRTAP* (*n* = 3, 4.05%), *P3H1* (*n* = 3, 4.05%), *SERPINH1* (*n* = 2, 2.70%), *SEC24D* (*n* = 3, 4.05%), and *PLOD2* (*n* = 1, 1.35%) respectively. Thus, *WNT1* represents the most frequent pathogenic gene of AR-OI in Chinese population. The most common clinical manifestations of AR-OI patients include walking problem (72.86%), scoliosis (65.28%) and frequent fractures (fractures ≥2/year) (54.05%). Interestingly, ptosis represents a unique phenotype of patients carrying *WNT1* variants, and it was rare in patients harboring other pathogenic genes. Our study expanded the mutation spectrum of AR-OI and enriched the knowledge of genotypic and phenotypic correlation in Chinese cohort with AR-OI.

## Introduction

Osteogenesis imperfecta (OI), also known as brittle bone disease, is a genetically and clinically heterogeneous skeletal disorder. Typical clinical manifestations include low bone mass, frequent fractures, short stature, blue sclerea, and bone deformity. More than 90% of OI cases are caused by a defect of type I collagen, and therefore OI is known as an autosomal dominant inherited disease due to a mutation in *COL1A1* or *COL1A2*, which encodes alpha 1 or alpha 2 chains of type I collagen (Marini et al., 2007). Multiple genes have been reported to contribute to the development of autosomal recessive OI (AR-OI), including *SERPINF1*, *LEPRE1*, *CRTAP*, *PPIB*, *FKBP10*, *BMP1*, *SP7*, *PLOD2*, *TMEM38B*, *P4HB*, *SERPINH1*, *SEC24D*, *WNT1*, *CREB3L1*, and *SPARC* (Kang et al., 2017). Although the incidence rate of AR-OI is < 10% of the whole OI population, the clinical manifestations of AR-OI are much more severe than the dominant OI patients (Liu et al., 2017; Li et al., 2019).

Synthesis of type I collagen is a sophisticated process. With the development of next generation sequencing, remarkable progress has been made in identifying new genes associated with the modification and folding of type I collagen. These genes have formed a unique biological network, and deficiency in any individual gene may lead to recessive OI. Propeptides encoded by *COL1A1* and *COL1A2* undergo post-translational modifications in the endoplasmic reticulum, followed by transportation to Golgi and cleavage of N-/C- terminal propeptides. Consequently, collagen fibers are formed to further build up the collagen matrix. Defect in any step during the type I collagen synthesis can lead to the development of OI (Kang et al., 2017). For example, deficiency of *CRTAP*, *P3H1* or *PPIB*, the components of collagen prolyl 3-hydroxylation complex, will lead to defects in post translational modification of unfolded collagen alpha-chains (Morello et al., 2006). Mutations in *FKBP10*, *SERPINH1* or *BMP1* cause defects in collagen folding and crosslinking (Koide et al., 2006; Schwarze et al., 2013; Kang et al., 2017). Alterations in *SP7* (Azetsu et al., 2017), *WNT1* (Keupp et al., 2013; Laine et al., 2013; Pyott et al., 2013) or *CREB3L1* (Murakami et al., 2009) affect osteoblast differentiation (Forlino and Marini, 2016). Dysfunction of PEDF, which is encoded by *SERPINF1*, will damage the bone homeostasis (Akiyama et al., 2010).

Although mutation spectrums on autosomal dominant OI have been established in large cohorts of Chinese (Li et al., 2019), Swedish (Lindahl et al., 2015) and Canadian/American populations (Bardai et al., 2016), mutation spectrum on AR-OI remains unclear due to its rare incidence. There was only one report about the molecular spectrum based on findings from a small cohort of 19 recessive OI patients from Mediterranean (Rauch et al., 2010). Here, we examined the mutation spectrum in 74 AR-OI families, the largest cohort worldwide to our knowledge. Current findings expand our knowledge about the gene spectrum and phenotypic spectrum of AR-OI, and provide important information for genetic diagnosis of AR-OI patients.

## Materials and Methods

### Subjects

We recruited 74 AR-OI probands from a cohort of 1095 OI patients (646 families) in mainland China. These patients displayed typical clinical manifestations of OI: recurrent fractures, short stature, bone malformation, with or without extra-skeletal manifestations such as blue sclera, hearing loss and dentinogenesis imperfecta. After obtaining approval from Institutional Review Board (IRB) of the Institute of Basic Medical Sciences, Chinese Academy of Medical Sciences, Beijing, China (015-2015) and informed consent from all participants/legal guardians of children under 18, peripheral blood samples were collected from all available family members of AR-OI patients. Skin samples were collected from some of the patients based on the availability.

Clinical parameters of probands were recorded at their first visit, including age, gender, height, times of fractures, presence of ptosis, scoliosis, blue sclerae, dentinogenesis imperfecta, and ability of walking. Height was also converted to Z-score calculated based on the age and medium height of Chinese population (Li et al., 2019).

### Variant Nomenclature

Variants were named according to the nomenclature provided by Human Genome Variation Society^1^. Genomic DNA and cDNA sequences of *SERPINF1* (NC_000017.10), *CRTAP* (NC_000003.11), *SERPINH1* (NC_000011.9), *FKBP10* (NC_000017.10), *PLOD2* (NC_000003.11), *WNT1* (NC_000012.11), *SEC24D* (NC_000004.11), *P3H1* (NC_000001.11) were obtained from National Center for Biotechnology Information (NCBI) reference sequence and University of California, Santa Cruz (UCSC) Genome browser database^2^.

### Whole Exome Sequencing (WES)

Samples from 59 probands underwent WES process. Genomic DNA was extracted from leukocytes using a standard sodium dodecyl sulfate-proteinase K-phenol/chloroform extraction method. 1–3 μg genomic DNA was used for WES as described previously (Li et al., 2019). DNA was fragmented as 150 bp, and the primers and adapters were then ligated to the DNA fragments to construct libraries. The analysis was performed on singletons, looking on previously known genes as filtering criteria. Sequencing was carried out on HiSeq 4000 System (Illumina). SAMtools mpileup and bcftools were used for variant calling and SNP/Indels identification. Control-FREEC was utilized for CNV detection.

### Genomic Panel Sequencing

Fifteen probands were examined by genomic panel sequencing. Customized panel sequencing including 184 genes related to the monogenic disorders focused in our lab was conducted as described previously (Li et al., 2019). 21 OI-related genes were included with 18 recessive OI genes. Sequencing was performed on the HiSeq 2500 System (Illumina, San Diego, CA, United States). All reference sequences were based on the GRCh37/hg19 assembly of the human genome.

### Confirmation of Pathogenic Mutation by Sanger Sequencing

To verify the candidate mutations detected by WES or Panel Sequencing, genomic DNA was amplified following the PCR program: 95°C for 3 min; 94°C for 30 s, 58°C for 30 s, 72°C for 50 s (35 cycles); 72°C for 8 min. PCR amplified fragments was subjected to Sanger Sequencing based on Applied Biosystems 3730xl DNA Analyzer (Thermo Fisher Scientific, Waltham, MA, United States). Sequence was analyzed using CodonCode Aligner (version 6.0.2.6; CodonCode, Centerville, MA, United States).

### Isolation and Culturing of Dermal Fibroblasts

To test the splicing effect caused by two variants in *FKBP10* in proband PUMC-121 on endogenous level, skin samples of the proband and an ethnically matched healthy individual were collected from fresh skin biopsies. Human fibroblasts were maintained at 37°C and 5% CO_2_ and supplied with F-12 supplemented with 1% L-glutamine, 20% fetal bovine serum, 1% sodium pyruvate, and 1% penicillin-streptomycin.

### RNA Level Analysis

When a mutation was located in an intron, RNA level analysis would be conducted. Total RNA was isolated from peripheral blood or skin fibroblasts using Trizol reagent (Invitrogen, Cat. No. 15596018) followed by cDNA preparation using GoScript^TM^ Reverse Transcription System (Promega, Cat. No. A5001), according to the manufacturer’s instructions. cDNA was used for sequencing or for quantitative PCR analysis.

### Quantitative PCR (qPCR)

qPCR was carried out to detect large fragment deletion and mRNA expression level of *FKBP10* gene. PCR amplification was carried out with SYBR Premix ExTaq (Takara, Bio., Dalian, China) and primer pairs (Supplementary Table S1) according to the manufacturer’s protocol. The reactions were monitored continuously in a Rotor-Gene 6000 instrument (Qiagen, Hilden, Germany) according to the following program: 95°C for 3 min followed by 40 cycles of 95°C for 10 s, 60°C for 15 s, and 72°C for 20 s. The relative copy number (RCN) of the targeted sequence was normalized to the expression levels of GAPDH by calculating the ΔCt (Ct_gene of interest_ - Ct_GAPDH_), and RCN = 2^–ΔΔCt^.

### T-Clone Sequencing

T-Clone sequencing was used when Sanger sequencing results showed interlaced alleles. Briefly, The PCR product of target samples with disrupted signals was linked to the pMD19-T vector. The ligation reaction contained 4 μl Solution I (Takara, Shiga, Japan), 1 μl pMD19-T vector and 5 μl purified PCR product. DNA Sanger sequencing was performed followed by vector ligation, *E. coli* transformation and bacterial culturing.

### Statistical Analysis

Statistical analysis was conducted for age of first onset, times of fractures, frequency of fractures, height, height Z-score and ptosis for the patients with pathogenic genes *WNT1*, *SERPINF1*, and *FKBP10*. GraphPad Prism (version 6.00; GraphPad Software, La Jolla, CA, United States) was used for statistical analysis and was performed for *n* ≥ 9. Differences between three or more groups were analyzed by one-way ANOVA, and comparisons between two groups were analyzed by student *t*-test. Graphs were presented as average ± standard deviation, and *p* < 0.05 was considered as significant difference.

## Results

### Phenotypic Characterization of AR-OI

**FIGURE 1:**
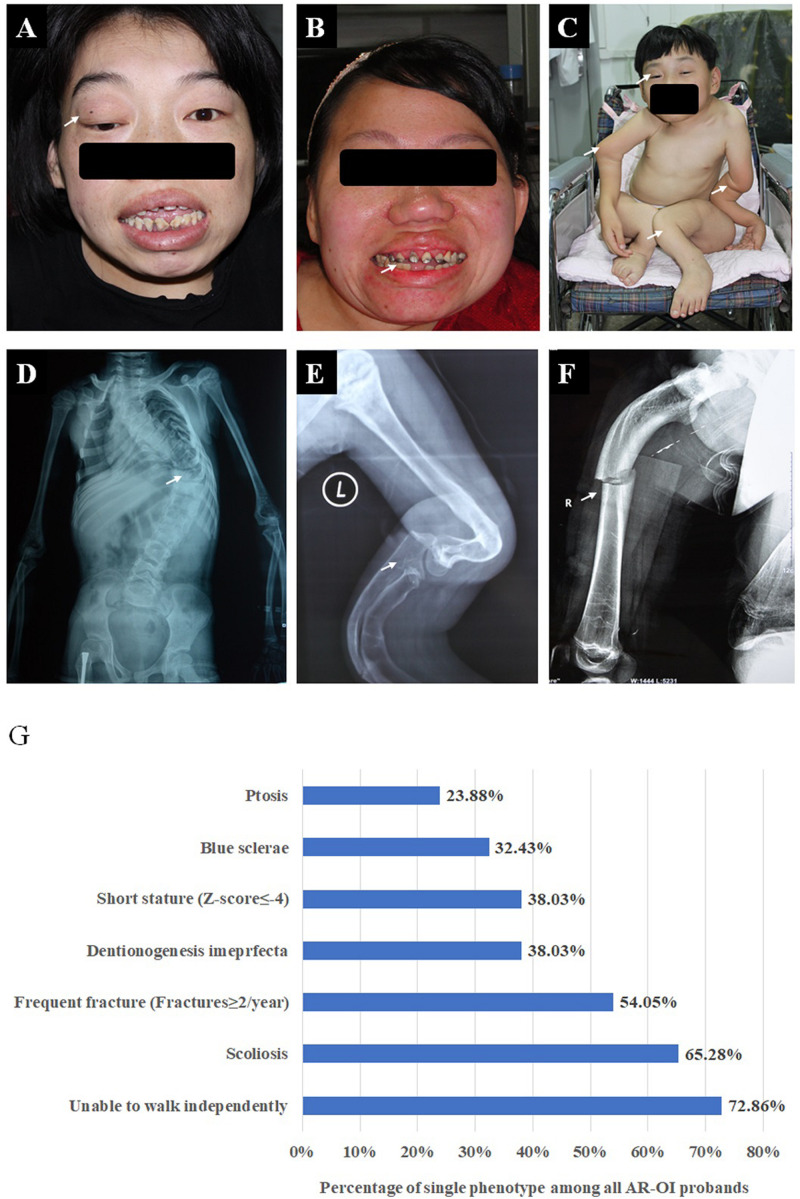
Phenotypic characteristics of AR-OI patients **(A–F)**. AR-OI patients had severe clinical manifestations including ptosis **(A)**, dentinogenesis imperfecta **(B)**, severe limb deformity and disability to walk independently **(C)**, scoliosis **(D)**, skeleton deformity **(E)**, and frequent fractures **(F)**. **(G)** Percentage of the presence of each clinical manifestation in AR-OI probands.

We recruited 74 probands (39 males, 34 females, and one gender unknown fetus) with AR-OI from 60 non-consanguineous families and 14 consanguineous families. Except for the fetus, their ages ranged from 6 months to 35 years old and their parents were confirmed to be unaffected. The clinical manifestations of the 74 probands, including frequent fractures, scoliosis, short stature, blue sclerae, ptosis, disability to walk and dentinogenesis imperfecta (DI) were recorded and analyzed (Figure 1). Phenotypes of all probands were summarized in Supplementary Table S2. These patients presented severe phenotypes: most of them showed disability to walk (72.86%, Figure 1C) and scoliosis (65.28%, Figure 1D). Nearly half of them experienced more than 2 fracture times per year (54.05%, Figure 1F), 38.03% of them had dentinogenesis imperfecta (Figure 1B), and 38.03% of patients presented extremely low short statures with a Z score less than −4 SD (corresponding to type III OI). Compared to dominant inherited OI, blue sclerae was less frequent (32.43%) in AR-OI individuals. A unique phenotype, ptosis (23.88%), was observed in recessive inherited OI patients (Figure 1A), but was absent in dominant OI patients. None of AR-OI individuals displayed hearing loss or intellectual disability.

### Genotypic Characterization of Recessive OI

#### Variants Identification of Recessive OI

**FIGURE 2:**
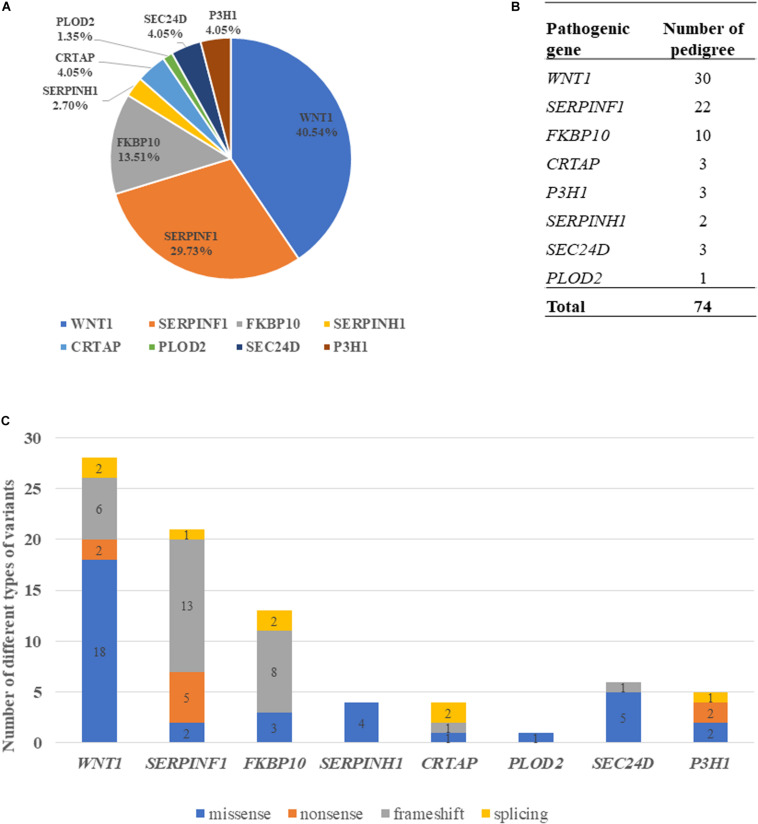
Genotypic characterization of AR-OI patients. **(A)** Mutation distribution in Chinese AR-OI patients. **(B)** The number of pedigrees in each pathogenic gene. **(C)** Distribution of mutation types in Chinese AR-OI patients.

Variants of the 74 probands were identified in *WNT1* (*n* = 30), *SERPINF1* (*n* = 22), *FKBP10* (*n* = 10), *SERPINH1* (*n* = 2), *CRTAP* (*n* = 3), *PLOD2* (*n* = 1), *P3H1* (*n* = 3), and *SEC24D* (*n* = 3) (Table 1 and Figure 2B). Mutations in *WNT1* showed the highest percentage (40.54%) in this cohort, followed by mutations in *SERPINF1* (29.73%) and *FKBP10* (13.51%, Figure 2A).

**TABLE 1:** Identification of mutations of recessive osteogenesis imperfecta in Chinese population.

**Proband number**	**Family history**	**Consan- guineous**	**Allele origin**	**Variant type**	**Variant position**	**Nucleotide change**	**Amino acid change**	**Novelty**
***WNT1***								
PUMC-3 (*n* = 2)	Y	N	Paternal; maternal	Missense; missense	Exon 3; exon 4	c.397G > A; c.677C > T	p.Ala133Thr; p.Ser226Leu	R; R
PUMC-10	N	N	Paternal; maternal	Frameshift; frameshift	Exon 3; exon 3	c.506dupG; c.506dupG	p.Cys170Leufs*6; p.Cys170Leufs*6	R; R
PUMC-21	N	N	Paternal; maternal	Frameshift; frameshift	Exon 3; exon 3	c.506dupG; c.506dupG	p.Cys170Leufs*6; p.Cys170Leufs*6	R; R
PUMC-71	N	N	Paternal; maternal	Missense; missense	Exon 2; exon 3	c.301C > T; c.382T > G	p.Arg101Cys; p.Phe128Val	R; R
PUMC-128 (*n* = 2)	Y	N	*De novo*; maternal	Nonsense; missense	Exon 2; exon 3	c.189delG; c.620G > A	p.Leu64*; p.Arg207His	R; R
PUMC-145	N	Y	Paternal; maternal	Missense; missense	Exon 4; exon 4	c.677C > T; c.677C > T	p.Ser226Leu; p.Ser226Leu	R; R
PUMC-154	N	N	Paternal; maternal	Frameshift; frameshift	Exon 3; exon 3	c.466delC; c.466delC	p.Arg156Glyfs*43; p.Arg156Glyfs*43	R; R
PUMC-158	N	N	Paternal; maternal	Frameshift; missense	Exon 2; exon 3	c.216dupA; c.506G > A	p.Arg73Thrfs*82; p.Gly169Asp	R; R
PUMC-217	N	N	Paternal; maternal	Missense; missense	Exon 3; exon 3	c.620G > A; c.620G > A	p.Arg207His; p.Arg207His	R; R
PUMC-221 (*n* = 2)	Y	N	Paternal; maternal	Frameshift; frameshift	Exon 3; exon 3	c.506dupG; c.506dupG	p.Cys170Leufs*6; p.Cys170Leufs*6	R; R
PUMC-226	N	N	Paternal; maternal	Missense; missense	Exon 3; exon 3	c.620G > A; c.620G > A	p.Arg207His; p.Arg207His	R; R
PUMC-230	N	N	Maternal; paternal	Missense; nonsense	Exon 2; exon 4	c.301C > T; c.681C > A	p.Arg101Cys; p.Cys227*	R; R
PUMC-245	N	N	Maternal; paternal	Splicing; missense	Intron 1; exon 4	c.105-2A > G; c.674G > T	p.?; p.Gly225Val	R; R
PUMC-247	N	Y	Paternal; maternal	Missense; missense	Exon 4; exon 4	c.677C > T; c.677C > T	p.Ser226Leu; p.Ser226Leu	R; R
PUMC-258	N	N	Paternal; maternal	Frameshift; missense	Exon 1; exon 4	c.10delT; c.677C > T	p.Trp4Glyfs*35; p.Ser226Leu	R; R
PUMC-274	N	N	Maternal; paternal	Missense; missense	Exon 2; exon 4	c.301C > T; c.937C > T	p.Arg101Cys; p.Arg313Cys	R; R
PUMC-277	N	N	Paternal; maternal	Missense; frameshift	Exon 1; exon 2	c.2T > C; c.216dupA	p.?; p.Arg73Thrfs*82	R; R
PUMC-281 (*n* = 3)	Y	N	Maternal; paternal	Missense; nonsense	Exon 4; exon 4	c.770T > C; c.774C > A	p.Leu257Pro; p.Try258*	R; R
PUMC-327	Y	N	Paternal; maternal	Splicing; missense	Intron 1; exon 3	c.104 + 1G > A; c.501G > C	p.?; p.Trp167Cys	R; R
PUMC-329	N	N	Paternal; maternal	Missense; missense	Exon 3; exon 3	c.371C > T; c.620G > A	p.Thr124Met; p.Arg207His	R; R
PUMC-471 (*n* = 2)	Y	Y	Paternal; maternal	Missense; missense	Exon 4; exon 4	c.677C > T; c.677C > T	p.Ser226Leu; p.Ser226Leu	R; R
PUMC-490	N	N	Paternal; maternal	Missense; missense	Exon 3; exon 3	c.620G > A; c.620G > A	p.Arg207His; p.Arg207His	R; R
PUMC-491	N	Y	Paternal; maternal	Missense; missense	Exon 4; exon 4	c. 557A > T; c. 557A > T	p.Asp186Val; p.Asp186Val	N; N
PUMC-554	N	N	Paternal; maternal	Missense; missense	Exon 4; exon 4	c.677C > T; c.677C > T	p.Ser226Leu; p.Ser226Leu	R; R
PUMC-555	N	N	Paternal; maternal	Missense; missense	Exon 3; exon 4	c.397G > A; c.986G > A	p.Ala133Thr; p.Cys329Thr	R; N
PUMC-572 (*n* = 2)	Y	N	Paternal; maternal	Missense; missense	Exon 2;exon 4	c.301C > T; c.710C > A	p.Arg101Cys; p.Pro237His	R; N
PUMC-586 (*n* = 2)	Y	N	Paternal; maternal	Missense; missense	Exon 3; exon 4	c.493T > C; c.677C > T	p.Trp165Arg; p.Ser226Leu	N; R
PUMC-612	N	N	Paternal; maternal	Missense; missense	Exon 4; exon 4	c.677C > T; c.677C > T	p.Ser226Leu; p.Ser226Leu	R; R
PUMC-618	N	N	Maternal; paternal	Missense; missense	Exon 3; exon 3	c.514G > T; c.590T > C	p.Asp172Tyr; p.Leu197Pro	N; N
PUMC-630	N	N	Paternal; maternal	Missense; frameshift	Exon 3; exon 3	c.397G > A; c.505_506del	p.Ala133Thr; (p.Gly169Leufs*6)	R; N
***SERPINF1***								
PUMC-4	Y	N	Maternal; paternal	Frameshift; frameshift	Exon 2; exon 6	c.261_265 dupGGCCC; c.879delC	p.Leu89Argfs*26; p.Thr294Profs*8	R; R
PUMC-33 (*n* = 2)	Y	Y	Paternal; maternal	Inframe; inframe	Exon 2; exon 2	c.271_279dup; c.271_279dup	p.Ala91_Ser93dup; p.Ala91_Ser93dup	R; R
PUMC-74	N	Y	Paternal; maternal	Missense; missense	Exon 1; exon 1	c.1A > G; c.1A > G	p.?; p.?	R; R
PUMC-150 (*n* = 2)	Y	N	Maternal; paternal	Frameshift; nonsense	Exon 2; exon 3	c.248_249delTC; c.397C > T	p.Leu83Glnfs*28; p.Gln133*	R; R
PUMC-255 (*n* = 2)	Y	Y	Paternal; maternal	Nonsense; nonsense	Exon 6; exon 6	c.907C > T; c.907C > T	p.Arg303*; p.Arg303*	R; R
PUMC-275	N	N	Paternal; maternal	Inframe; inframe	Exon 6; exon 6	c.829_831 delTTC; c.829_831 delTTC	p.Phe277del; p.Phe277del	R; R
PUMC-306	N	Y	Paternal; maternal	Frameshift; frameshift	Exon 2; exon 2	c.261_265 dupGGCCC; c.261_265 dupGGCCC	p.Leu89Argfs*26; p.Leu89Argfs*26	R; R
PUMC-331	N	N	Paternal; maternal	Frameshift; frameshift	Exon 1; exon 1	c.77dupC; c.77dupC	p.Glu27Glyfs*38; p.Glu27Glyfs*38	R; R
PUMC-381	N	N	Paternal; maternal	Splicing; frameshift	Intron 2; exon 4	c.283 + 1G > T; c.498-499delCA	p.?; p.Arg167Serfs*35	R; R
PUMC-348	N	N	Paternal; maternal	Frameshift; frameshift	Exon 7; exon 7	c.1193dupT; c.1193dupT	p.Arg399Glufs*31; p.Arg399Glufs*31	N; N
PUMC-422	N	N	Paternal; maternal	Frameshift; frameshift	Exon 6; exon 6	c.839dupT; c.839dupT	p.Lys281Glufs*20; p.Lys281Glufs*20	N; N
PUMC-496	N	N	Paternal; maternal	Frameshift; frameshift	Exon 1; exon 1	c.77dupC; c.77dupC	p.Glu27Glyfs*38; p.Glu27Glyfs*38	R; R
PUMC-482	N	N	Paternal; maternal	Frameshift; frameshift	Exon 2; exon 5	c.271_279del; c.748_763del	p.Ala91_Ser93del; p.Val250Trpfs*14	N; N
PUMC-495	N	N	Paternal; maternal	Frameshift; frameshift	Exon 1; exon 1	c.77dupC; c.77dupC	p.Glu27Glyfs*38; p.Glu27Glyfs*38	R; R
PUMC-413	N	N	Paternal; maternal	Nonsense; nonsense	Exon 6; exon 6	c.808G > T; c.808G > T	p.Gly270*; p.Gly270*	R; R
PUMC-527 (*n* = 3)	Y	Y	Paternal; maternal	Nonsense; nonsense	Exon 6; exon 6	c.907C > T; c.907C > T	p.Arg303*; p.Arg303*	R; R
PUMC-546	N	Y	Paternal; maternal	Frameshift; frameshift	Exon 6; exon 6	Chr17:1,679,402-1,680,079; Chr17:1,679,402-1,680,079 (hg19)	p.?; p.?	N; N
PUMC-585 (*n* = 2)	Y	N	Paternal; maternal	Nonsense; nonsense	Exon 4; exon 4	c.553C > T; c.553C > T	p.Gln185*; p.Gln185*	N; N
PUMC-595	N	N	Paternal; maternal	Frameshift; frameshift	Exon 6; exon 6	c.863_866 dupTGAT; c.879delC	p.Glu290Aspfs*12; p.Thr294Profs*8	N; R
PUMC-607	N	N	Paternal; maternal	Missense; frameshift	Exon 2; exon 7	c.167C > G; c.271_279dup	p.Ala56Gly; p.Ala91_Ser93dup	R; R
PUMC-611	N	Y	Paternal; maternal	Nonsense; nonsense	Exon 1; exon 1	c.79G > T; c.79G > T	p.Glu27*; p.Glu27*	R; R
PUMC-624	N	Y	Paternal; maternal	Nonsense; nonsense	Exon 3; exon 3	c.397C > T; c.397C > T	p.Gln133*; p.Gln133*	R; R
***FKBP10***								
PUMC-68 (*n* = 2)	Y	N	Paternal; maternal	Frameshift; frameshift	Exon 5; exon 5	c.809_843del; c.813_814delGA	p.Leu270Glnfs*91; p.Glu271Aspfs*101	R; R
PUMC-121	N	N	Paternal; maternal	Splicing; splicing	Intron 5; exon 6	c.918-6T > G; c.1016G > A	p.Ser306Argfs*18; p.Ser306Argfs*18	R; R
PUMC-157	N	N	Paternal; maternal	Missense; frameshift	Exon 2; exon 5	c.344G > A; c.831delC	p.Arg115Gln; p.Gly278Alafs*20	R; R
PUMC-207 (*n* = 2)	Y	N	Maternal; paternal	Frameshift; gross deletion	Exon 5	c.831delC; chr17: g.39974881 _39980318del (hg19)	p.Gly278Alafs*20; p.?	R; R
PUMC-405	N	N	Paternal; maternal	Frameshift; missense	Exon 2; exon 2	c.320_353del; c.344G > A	p.107_118del; p.Arg115Gln	N; R
PUMC-431	N	N	Maternal; paternal	Missense; frameshift	Exon 2; exon 5	c. 344G > A; c.831dupC	p.Arg115Gln; p.Gly278Argfs*95	R; R
PUMC-525	N	N	Maternal; paternal	Frameshift; frameshift	Exon 5; exon 8	c.831dupC; c.1390delG	p.Gly278Argfs*95; p.Glu464Argfs*67	R; N
PUMC-536	N	N	Maternal; paternal	Frameshift; frameshift	Exon 5; exon 5	c.831dupC; c.831dupC	p.Gly278Argfs*95; p.Gly278Argfs*95	R; R
PUMC-605	Y	Y	Paternal; maternal	Splicing; splicing	Intron 5; intron 5	c.918-3C > G; c.918-3C > G	p.?; p.?	R; R
PUMC-606	N	N	Maternal; paternal	Missense; splicing	Exon 1; intron 5	c.1A > G; c.918-6T > G	p.Met1Val; p.Ser306Argfs*18	N; R
***SERPINH1***								
PUMC-285	N	N	Maternal; paternal	Missense; missense	Exon 1; exon 4	c.149T > G; c.1214G > A	p.Leu50Arg; p.Arg405His	R; R
PUMC-324 (*n* = 2)	Y	N	Paternal; maternal	Missense; missense	Exon 1; exon 3	c.589G > C; c.800T > C	p.Gly197Arg; p.Leu267Pro	R; R
***CRTAP***								
PUMC-118 (*n* = 2)	Y	N	Paternal; maternal	Nonsense; splicing	Exon 1; intron 1	c.202G > T; c.471 + 4delA	p.Glu68*; p.?	R; R
PUMC-456 (*n* = 2)	Y	N	Paternal; maternal	Splicing; splicing	Intron 6; intron 6	c.1153-3C > G; c.1153-3C > G	p.Gly385Argfs*46; p.Gly385Argfs*46	N; N
PUMC-582	N	N	Paternal; maternal	Frameshift; splicing	Exon 1; intron 6	c.18_25 dupGG GGGCCG; c.1153-3C > G	p.Ala9Glyfs*7; p.Gly385Argfs*46	N; N
***PLOD2***								
PUMC-320	N	Y	Paternal; maternal	Missense; missense	Exon 18; exon 18	c.1856G > A; c.1856G > A	p.Arg619His; p.Arg619His	R; R
***P3H1***								
PUMC-566	N	N	Paternal; maternal	Nonsense; missense	Exon3; exon14	c.652G > T; c.1948G > C	p.Glu218*; p.Gly650Arg	N; N
PUMC-590	N	N	Maternal; paternal	Nonsense; nonsense	Exon3; exon15	c.652G > T; c.2164C > T	p.Glu218*; p.Gln722*	N; N
PUMC-597	N	N	Paternal; maternal	Missense; splicing	Exon 9; intron 14	c.1466T > C; c.1915-1G > A	p.Leu489Pro; p?	R; R
***SEC24D***								
PUMC-266 (*n* = 2)	Y	N	Maternal; paternal	Missense; missense	Exon 16; exon 21	c.2185G > A; c.2869A > G	p.Val729Met; p.Thr957Ala	N; N
PUMC-204	N	N	Maternal; paternal	Missense; missense	Exon 6; exon7	c.875C > T; c.938G > A	p.Pro292Leu; p.Arg313His	R; R
PUMC-514	N	N	Paternal; maternal	Frameshift; missense	Exon 1; exon17	c.113dupC; c.2296G > A	p.Thr39Asnfs*16; p.Gly766Ser	R; N

* For each proband, *n* = 1 unless stated on the table; *n* = number of patients; In lane Family history/Consanguineous, Y = Yes, N = No; In lane Novelty, R = Reported, N = Novel. * The novelty was recorded according to HGMD database (professional 2020.01).

Four major types of variants were found in this cohort, including missense, nonsense, frameshift and splicing mutations. We analyzed the number of each type of variant in each pathogenic gene and found that missense mutation was the main variant type in *WNT1*, *SERPINH1* and *SEC24D*, and frameshift was frequently observed in *SERPINF1* and *FKBP10* (Figure 2C). Most variants were located at exons, but a homozygous intronic variant c.1153-3C > G was found in *CRTAP* (PUMC-456) (Supplementary Figure S1). Sequencing analysis of RT-PCR product of RNA isolated from dermal fibroblasts of this proband and his father confirmed that variant c.1153-3C > G led to an insertion of AG in the mutant transcript (Supplementary Figure S1C).

#### Establishment of Mutation Spectrums of *WNT1*, *SERPINF1*, and *FKBP10*

**FIGURE 3:**
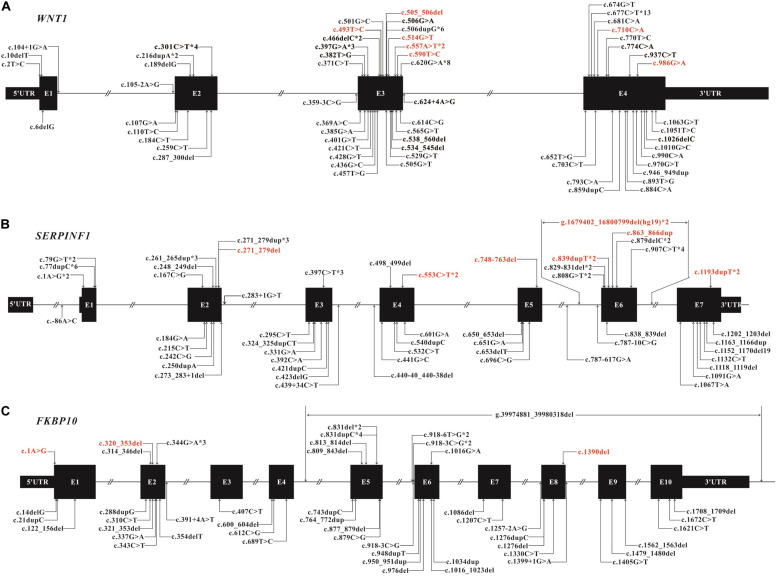
Mutation spectrum of *WNT1* **(A)**, *SERPINF1* **(B)**, and *FKBP10* **(C)** of Chinese AR-OI patients. Black boxes represent the exons in each gene and lines represent the introns. Variants found in this study are listed above the boxes with novel variants in red and reported variants in black. Variants that have been reported previously are also listed under the boxes. Notation *digit indicates the number of probands carrying the same variant.

We identified 82 variants including 25 novel variants and 57 HGMD (professional 2020.01) reported variants (Table 1). Note that there were 36 variants reported in our previous study (Li et al., 2019). Because most variants are located in *WNT1*, *SERPINF1* and *FKBP10*, the mutation spectrums of these genes were established (Figure 3).

##### *WNT*1

We found 28 variants in *WNT1* from 30 probands, including 21 reported variants and 7 novel variants (Table 1 and Figure 3A). There were some hotspot variants in *WNT1*: homozygous mutation c.506dupG(p.Cys170Leufs^∗^6) was found in three unrelated non-consanguineous families (PUMC-10, 21, and 221), leading to the production of truncated protein; Mutation c.677C > T(p.Ala133Thr) was observed in PUMC-3, 145, 247, 258, 471, and 554; Mutation c.620G > A (p.Arg207His) occurred in two probands (PUMC-128 and 329) and three non-consanguineous families (PUMC-217, 226, and 490), representing a hotspot in this Chinese cohort.

##### *SERPINF*1

Eight consanguineous and 14 non-consanguineous families were identified with mutations in *SERPINF1*, including 21 variants (14 reported variants and 7 novel variants) (Table 1 and Figure 3B). A known homozygous mutation, c.77dupC (p.Glu27Glyfs^∗^38), was found in three non-consanguineous families, which resulted in frameshift and truncated protein. Interestingly, there were three siblings from a consanguineous family (PUMC-527) identified with same homozygotes of a novel variant, c.907C > T in *SERPINF1*, but they exhibited extremely different phenotypes (Supplementary Figure S2). The proband had severe scoliosis and unable to walk independently with more than 45 times of fracture. However, his elder sister, harboring the same genotype, did not present any OI phenotype at all with only once fracture lifetime.

##### *FKBP1*0

Thirteen variants in *FKBP10*, including 10 known variants and 3 novel variants, were identified in 10 non-consanguineous families (Table 1 and Figure 3C). Hotspot c.831indelC was identified in PUMC-157, 207, 431, 525 and 536, and these variants led to a premature termination codon. Hotspot c.344G > A was identified in PUMC-157, 405, and 431. Among the patients with variants in *FKBP10*, three special cases needed to be highlighted. Case I, Patient PUMC-405 was a compound heterozygote with mutations in *FKBP10*. Sanger sequencing analysis by software *Chromas* showed disrupted signals in exon 2 in *FKBP10* (Supplementary Figure S4A), and two alleles were separated by T clone sequencing of the mutant region: c.320_353del(p.107_118del) with a 34 bp deletion (Supplementary Figure S4B), and a missense mutation c.344G > A(p.Arg115Gln) within the deleted region (Supplementary Figure S4C); Case II, Patient PUMC-207 harbored a gross deletion and an indel variant in *FKBP10*. The compound mutations found in proband PUMC-207 were inherited from the parents: the indel of c.831delC was inherited from the mother and the gross deletion of chr17: g.39974881_39980318del (hg19) was inherited from the father, which was confirmed in breakpoint analysis using Gap-PCR and Sanger sequencing (Supplementary Figure S5); Case III, Patient PUMC-121 was previously reported that an intronic variant c.918-6T > G and a variant in adjacent exon c.1016G > A in *FKBP10* similarly drove to skipping of exon 6, indicated by a minigene assay (Supplementary Figures S6A,B) (Li et al., 2019). Based on the sequencing results from cDNA (Supplementary Figure S6C) and RNA isolated from the patient’s dermal fibroblasts in the follow up study, we found that variant c.918-6T > G caused skipping of exon 6 and variant c.1016G > A caused partial skipping of exon 6. This was confirmed by qPCR analysis (Supplementary Figure S6D).

### Correlation Between Genotypic and Phenotypic Changes

**FIGURE 4:**
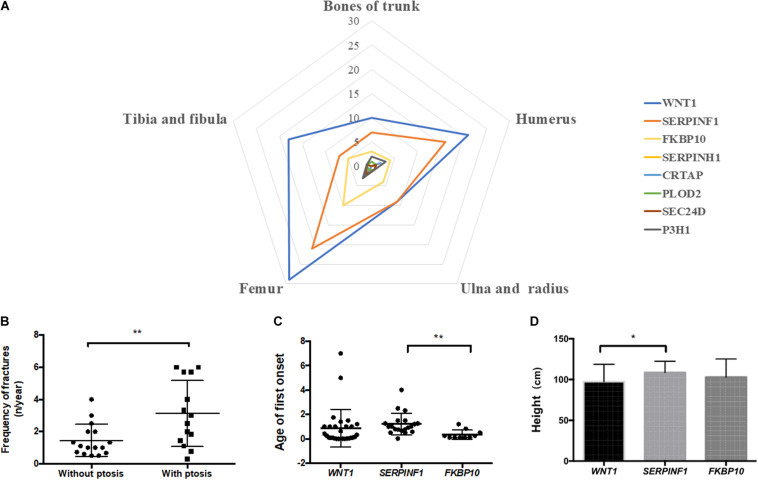
Correlation analysis of genotype and phenotype of Chinese AR-OI patients. **(A)** Analysis of fracture sites in patients with variants in *WNT1*, *SERPINF1*, *FKBP10*, *SERPINH1*, *CRTAP*, *PLOD2*, *P3H1*, and *SEC24D*. **(B)**. Fracture frequencies in patients with and without ptosis phenotype. Comparison of age of first onset **(C)** and height **(D)** of patients with variants in *WNT1*, *SERPINF1*, and *FKBP10* (*p < 0.05, **p < 0.01).

In order to answer whether there is a correlation between genotypes and observed phenotypes in AR-OI patients, we first analyzed the sites of fractures in patients harboring different pathogenic genes. We did not find significant difference in fracture locations among patients with different causative genes (Figure 4A). We then analyzed the phenotypes including fracture times, scoliosis, height, blue sclerae, DI, walking ability, and ptosis in these patients. Statistical analysis was conducted for *WNT1*, *SERPINF1* and *FKBP10*, with > 3 probands (Supplementary Table S3), and only ptosis was significantly different between groups.

#### Patients With *WNT1* Mutation Had Ptosis Phenotype

We noticed that AR-OI patients carrying *WNT1* mutations were prone to developing ptosis, with 46.67% penetrance (Supplementary Table S3). This manifestation was common in patients with a homozygous function loss of *WNT1*, but was rare in patients with a loss of *SERPINF1*. Ptosis distribution displayed significant difference in different genes (*p* = 0.0042). In patients with *WNT1* mutations, those with ptosis also showed more severe skeletal dysfunctions including higher fracture frequency (Figure 4B) and unable to walk independently (80%), as compared to those without ptosis.

#### Patients With Variants in *SERPINF1* Presented a Distinct Phenotype

Compared to patients with mutations in *WNT1* and *FKBP10*, patients with variants in *SERPINF1* showed significantly delayed first fracture age (*p* = 0.0072, Figure 4C) and taller height (*p* = 0.0460, Figure 4D).

## Discussion

With the development of next generation sequencing, genes associated with OI have been well-studied and identified. We examined mutation spectrum of AR-OI in a large cohort of 74 Chinese OI families. Current study unraveled 25 novel variants from 82 identified variants, and demonstrated *WNT1* mutation as the most frequent mutation in all AR-OI genes. Furthermore, these AR-OI patients showed a unique phenotype, ptosis.

Type I collagen is a heterotrimer structure with two alpha 1 chains and one alpha 2 chain. Dominant OI is caused by mutations in *COL1A1* or *COL1A2*, and phenotypes can vary from mild to moderate/severe depending on the whether the collagen defect was caused by haplo-insufficiency or helical mutations that led to dominant negative effect (Rauch et al., 2010; Lindahl et al., 2015). In AR-OI, alteration occurs during post translational modification progress when a triple helix pro-collagen is already formed, and hence most of these mutations affect both the synthesis of the whole type I collagen protein and bone homeostasis. Accordingly, AR-OI patients often exhibit more severe phenotypes than dominant OI, including significantly earlier first fracture age, higher fracture frequency, higher percentage of scoliosis and higher proportion of disability to walk (Li et al., 2019). Consistent with previous findings, more severe clinical manifestations were also observed in a cohort of Chinese patients with recessive OI in current study, including a high rate of scoliosis, disability of independent walking, extreme short stature, and high facture frequency (Figure 1G).

Current study in Chinese AR-OI patients suggests that *WNT1* is the most frequently involved gene, followed by *SERPINF1* and *FKBP10*. These findings differ from that in Caucasian population, which showed that *SERPINF1* was the highest, followed by *CRTAP* (Bardai et al., 2016). The most distinct phenotypic characteristic in Chinese AR-OI patients was a unique ptosis phenotype, which was presented almost exclusively in patients associated with *WNT1* variants (Supplementary Table S3). This result is consistent with recent finding that ptosis was a hallmark for OI patients with *WNT1* mutations from Indian and Turkish families (Nampoothiri et al., 2019), and that from seven Chinese families (Lu et al., 2018). *WNT1* signaling was critical for the cross talk between osteoblastic lineage and osteoclastic-lineage cells (Laine et al., 2013). In addition to its vital importance in bone homeostasis (Luther et al., 2018), *WNT1* also plays an essential role in neurological development (Faqeih et al., 2013; Aldinger et al., 2016). Because patients displayed ptosis all suffered neurological dysfunctions (Nampoothiri et al., 2019), it was postulated that ptosis phenotype may be associated with impaired brain or nerve development. Although we did not observe any intellectual problem in AR-OI patients with ptosis phenotype, these patients developed more severe skeletal phenotypes than those without ptosis (Figure 4D). No correlation was found between ptosis and mutation type or variant location. The mechanism of the development of ptosis in patients with *WNT1* variant-induced AR-OI remains to be elucidated in future studies.

The severity of skeletal phenotype was compared in patients with *WNT1*, *SERPINF1*, and *FKBP10* variants. Patients with variants in *SERPINF1* had the highest number of average fracture times (30.77 ± 6.626) and highest frequency of fractures (4.193 ± 0.7986 n/year). However, their stature is the tallest (108.7 ± 3.154 cm, Supplementary Table S3). Mrosk et al. (2018) reported that the phenotypic severity of genes associated with OI is: *WNT1* > *SERPINF1* > *FKBP10*, based on findings from 50 OI families, including 24 recessive OI families. In the current study, patients with *WNT1* variants presented the shortest height (97.40 ± 3.889) and highest percentage of scoliosis 80.00%, but did not show any significant difference in other parameters such as height Z-score, blue sclerae, dentinogenesis imperfecta and disability of independent walking from patients with variants in *SERPINF1* and *FKBP10* (Supplementary Table S3). It needs to be noted that patients with *FKBP10* variants all presented a short neck phenotype and severe spinal malformation (Supplementary Figure S3). FKBP10 is known to function as a molecular chaperone that interacts with collagen (Ishikawa et al., 2008), but how it affects the cervical vertebra development remains unclear.

Recurrent variants were found in *WNT1* (c.506dupG, c.677C > T, and c.620G > A), *SERPINF1* (c.77dupC, c.907C > T) and *FKBP10* (c.831indelC and c.344G > A). All the homozygous of these recurrent variants corresponded to a severe phenotype (Supplementary Table S2). In particularly, OI patients with the novel variant c.907C > T in *SERPINF1* presented the most severe phenotypes: with 45–100 times of fractures, severe scoliosis and extremely short stature (Z-score < −8) (Supplementary Table S2). Previous studies also identified some recurrent variants, such as c.506dupG in *WNT1*, but did not find a clear correlation with the severity of phenotype (Liu et al., 2016). Variant c.506dupG in *WNT1* was found to be a hotspot region in 5 families among 10 total families examined (Nampoothiri et al., 2019). Kelley et al. (2011) reported the recurrent variant, c.344G > A in *FKBP10* from a Caucasian child, who presented multiple fractures and a short stature. Further studies are needed to establish a causal-relationship between hotspot variant sites and phenotypes in these patients.

Frameshift occupied a high proportion in *SERPINF1* and *FKBP10* mutations (Figure 2C), suggesting that the two genes may have a distinct mechanism for OI development. In particular, most of variants located in *SERPINF1* may be involved in occurrence of nonsense/frameshift mediated pre-termination codon (Table 1), and these patients had very severe skeletal phenotypes, which is in line with previous findings (Becker et al., 2011). *SERPINF1* encodes pigment epithelium-derived factor (PEDF), which can inhibit osteoclastogensis by regulating osteoprotegerin expression (Akiyama et al., 2010) and enhancing pre-osteoblast differentiation (Toru Akiyama et al., 2003). The truncated PEDF protein which mainly results in the absence of C terminal of PEDF, led to the malfunction of protein (Shao et al., 2003). Therefore, nonsense/frameshift mutations in *SERPINF1* may lead to truncated PEDF and severe OI phenotype.

In summary, we examined the largest cohort (*n* = 74) of Chinese AR-OI probands and established the mutation spectrum of the three most frequent pathogenic genes (*WNT1*, *SERPINF1*, *FKBP10*) in this population. We also identified 25 novel variants and 7 hotspot variants. Genotypic and phenotypic analysis unraveled ptosis as a unique phenotype in patients with *WNT1* variants. Furthermore, patients with ptosis or hotspot variants showed more severe skeletal phenotypes than others. Current findings may enrich our understanding of the genetic basis of AR-OI, and our study provides new knowledge for a precise diagnosis of this disease in Chinese population.

## Data Availability Statement

The datasets for this article are not publicly available due to concerns regarding participant/patient anonymity. Requests to access the datasets should be directed to the corresponding author.

## Ethics Statement

The studies involving human participants were reviewed and approved by Institutional Review Board (IRB) of the Institute of Basic Medical Sciences, Chinese Academy of Medical Sciences, Beijing, China (015-2015). Written informed consent to participate in this study was provided by all participants/legal guardians of children under 18.

## Author Contributions

SL, YC, and HW performed the experiment and wrote the manuscript. XR and YW collected the samples of patients. HM, YG, FZ, LL, BM, TY, YYo, XG, and YYa conducted data analysis. XZho and XZhn designed and supervised this research. All authors performed critical reading and approved the final version of manuscript.

## Conflict of Interest

The authors declare that the research was conducted in the absence of any commercial or financial relationships that could be construed as a potential conflict of interest.

## Footnotes

1 http://www.hgvs.org/munomen

2 http://genome.ucsc.edu/

## Supplementary Material

FIGURE S1 Dermal fibroblast analysis confirmed the sequencing result of intronic variant c.1153-3C > G in *CRTAP*. **(A)** Pedigree information was shown in the family tree. **(B)** Results from genomic DNA sequencing showed an intronic mutation of c.1153-3C > G in *CRTAP* in the proband II1. Parents of the proband were heterozygous carrier of the same variant. **(C)** Sequencing of dermal fibroblasts of I1 and II1 confirmed an insertion of AG in the mutant transcript.

FIGURE S2 A consanguineous family that had the same variant but presented variable expressivity. **(A)** The pedigree of consanguineous family PUMC-527. **(B)** A photo of patients II1 (mild) and II2 (severe) with a wide difference in their phenotypes. **(C)** Genotype of all the familial members revealed by DNA sequencing.

FIGURE S3 Recessive OI patients with variants in *FKBP10* exhibited a unique short neck phenotype **(A–C)**. Photo records and X-ray examinations of three individuals with *FKBP10* variants. **(A)** Short neck (PUMC-68), **(B)** short neck and severe deformity of spinal (PUMC-68), **(C)** compressible vertebrae (PUMC-121).

FIGURE S4 A case of micro-deletion and missense mutation in the same region in *FKBP10*. **(A)** Sanger DNA sequencing of proband PUMC-405 showed disrupted signal in exon 2 in *FKBP10*. Two mutations was separated after T-clone sequencing: c.320_353del(p.107_118del) **(B)** and c.344G > A(p.Arg115Gln) which was located within the deletion region **(C)**.

FIGURE S5 A case of gross deletion in *FKBP10*. **(A)** Sanger sequencing results of the proband and his parents. **(B)** An intragenic deletion was found in the proband and his father indicated by quantitative real-time PCR. The values presented as triplicate determinations ± SD. RCN, relative copy number. **(C)** Breakpoint analysis showed that breakpoint was found in proband and his father (640 bp), while absence in the mother of proband because of the existence of original fragment. M: Marker, 640 bp: fragment length of the deletion junction product, 304 bp: control. **(D)** Sequence chromatograms of the g.41818632_41824069del breakpoint.

FIGURE S6 Identification of the splicing effect of compound mutations in *FKBP10*. **(A)** Sanger sequencing indicated a missense variant c.1016G > A(p.Arg339Gln) in exon 6 and an intronic variants c.918-6T > G in adjacent intron 5 in *FKBP10* in patient PUMC-121. **(B)** Minigene assay showed that both c.918-6T > G and c.1016G > A led in skipping of exon 6. **(C)** Sequencing result of RT-PCR product from RNA extracted from the patient’s dermal fibroblasts. **(D)** Relative normalized expression of exon 6, exons 4–5, and exons 7–8 comparing the patient and control. Gene expression in normal control was normalized as 1.
