# Supplementary material for: Genotypic and Phenotypic Analysis in Chinese Cohort With Autosomal Recessive Osteogenesis Imperfecta
Source: Front Genet. 2020 Sep 15;11:984. doi: 10.3389/fgene.2020.00984 (PMC7523636; doi:10.3389/fgene.2020.00984)
Supplement: FIGURE S1 — Dermal fibroblast analysis confirmed the sequencing result of intronic variant c.1153-3C > G in CRTAP. (A) Pedigree information was shown in the family tree. (B) Results from genomic DNA sequencing showed an intronic mutation of c.1153-3C > G in CRTAP in the proband II1. Parents of the proband were heterozygous carrier of the same variant. (C) Sequencing of dermal fibroblasts of I1 and II1 confirmed an insertion of AG in the mutant transcript. [file Data_Sheet_1.docx]

**Table S1. Primers used in this study.**

| **Gene name** | **Primer name** | **Sequence (5' → 3')** | **T_m_ (°C)** | **Size (bp)** |
| --- | --- | --- | --- | --- |
| *SERPINF1* | *SERPINF1*-5'UTR-F | TGCAAATGTGTGCGCCTTAG | 58 | 357 |
|  | *SERPINF1*-5'UTR-R | ACCCCTCTTTTCTGCTCCCT |  |  |
|  | *SERPINF1*-E1F | CATCAAGCTGAGGGTCCACT | 58 | 346 |
|  | *SERPINF1*-E1R | TGTCCTGTTTCCCGTGCTG |  |  |
|  | *SERPINF1*-E2F | TCCACAGAGAGCTCATGCGT | 58 | 487 |
|  | *SERPINF1*-E2R | CCTCGCTGAACTGAAATGTC |  |  |
|  | *SERPINF1*-E3F | TGAAAATTCCTTGGCCACCT | 58 | 340 |
|  | *SERPINF1*-E3R | AGGCAACTTGGGCTGCAAAG |  |  |
|  | *SERPINF1*-E4F | AGGCTCTCAAAGACGGGATG | 58 | 368 |
|  | *SERPINF1*-E4R | TGCACTCCAGTTAGGACTACAG |  |  |
|  | *SERPINF1*-E5F | CAGCTAAGCTCCCTTGAGTG | 58 | 376 |
|  | *SERPINF1*-E5R | AGCAGGCGACATGTAGACAG |  |  |
|  | *SERPINF1*-E6F | GGATGAAGGACGAGACCAGG | 58 | 423 |
|  | *SERPINF1*-E6R | TGTGGAGCCCTTGCGTTCT |  |  |
|  | *SERPINF1*-E7F | TAAATGCTCCTGGGCAAGTC | 58 | 643 |
|  | *SERPINF1*-E7R | GGCTAGAAGTAGAGGACCACC |  |  |
| *CRTAP* | *CRTAP*-E1F | ATCAGCCTGCGTGAACCA | 58 | 815 |
|  | *CRTAP*-E1R | TCTAGAACTGGAGGGGCAAC |  |  |
| *SERPINH1* | *SERPINH1*-5'UTR-F | AGGAAGCTCGCACTCTGAAG | 58 | 479 |
|  | *SERPINH1*-5'UTR-R | GGACTCCAATTTGTAGGCTCT |  |  |
|  | *SERPINH1*-E1F | TGGCAGCTCTGAGTCAAGTC | 58 | 905 |
|  | *SERPINH1*-E1R | TGAGAGGCAGTGAAGAATGG |  |  |
|  | *SERPINH1*-E2-3F | ATGGAGCAAAGACCTGTGGC | 58 | 740 |
|  | *SERPINH1*-E2-3R | CATCATCTTCCCAAGGGCAT |  |  |
|  | *SERPINH1*-E4F | CTGGCATACAGAAGGTGACC | 58 | 1231 |
|  | *SERPINH1*-E4R | CAGTAGCGAGTCTGCCTTAAC |  |  |
| *FKBP10* | *FKBP10*-E1F | CTCCGGAAAAAGTCATTGAA | 58 | 793 |
|  | *FKBP10*-E1R | AGGGTATCTTTGGGGAGATG |  |  |
|  | *FKBP10*-E2F | GAAGTGTGTGTGCGTATCCA | 58 | 430 |
|  | *FKBP10*-E2R | GCTGCCACTCTTCATACTGC |  |  |
|  | *FKBP10*-E3-4F | GTCTGGCCCCCTATCTCTTA | 58 | 701 |
|  | *FKBP10*-E3-4R | CCCTCGTGCAGTATACAACC |  |  |
|  | *FKBP10*-E5-6F | CTCTGGAGAGTGGGGCTAGT | 58 | 703 |
|  | *FKBP10*-E5-6R | GCATGCATGTTTTTGTGATG |  |  |
|  | *FKBP10*-E7-8F | TTGACTCTGGACAAACATCC | 58 | 1084 |
|  | *FKBP10*-E7-8R | AGGTTGCAGTGAGCTGAGAG |  |  |
|  | *FKBP10*-E9-10F | GGAGTCAGGAATGCCTTCAG | 58 | 997 |
|  | *FKBP10*-E9-10R | TGTCAGCAGGTCAGTCCCAC |  |  |
| *PLOD2* | *PLOD2*-E1F | AAATAAAATCACGGCCTTCC | 58 | 1072 |
|  | *PLOD2*-E1R | CGAGAACTCCAAGGAGTCAA |  |  |
|  | *PLOD2*-E2F | AAGGTTCATCATTTGGCAGT | 58 | 394 |
|  | *PLOD2*-E2R | CCAAATGCTATCTTCCTTGTG |  |  |
|  | *PLOD2*-E3F | CATTTTGGGGATGGATAACA | 58 | 540 |
|  | *PLOD2*-E3R | GACAGGGTTTCACCATCTTG |  |  |
|  | *PLOD2*-E4F | TGTGTAAATAGTTGGCACAGG | 58 | 468 |
|  | *PLOD2*-E4R | TTTAGGAACACCAACTCACA |  |  |
|  | *PLOD2*-E5F | TCTTTCATGGTGAGCTGTGA | 58 | 438 |
|  | *PLOD2*-E5R | ATCCAGCCAGGTGACATAAA |  |  |
|  | *PLOD2*-E6F | TTGCAACTATCGCAGTTTCT | 58 | 469 |
|  | *PLOD2*-E6R | GCCAGAAGCTTATCTTTTTCAC |  |  |
|  | *PLOD2*-E7F | GCTGGCTTGGAGTAGATGAA | 58 | 502 |
|  | *PLOD2*-E7R | ACGCCAAATGTGCTGTAAAT |  |  |
|  | *PLOD2*-E8F | TGGAGAAGACTATTCAGTGTGC | 58 | 422 |
|  | *PLOD2*-E8R | AGCAAATGGCTGAATCATCA |  |  |
|  | *PLOD2*-E9F | TAGAGAACTGCCATGGAGCA | 58 | 483 |
|  | *PLOD2*-E9R | GCAGTTTCATGTGGCTCATA |  |  |
|  | *PLOD2*-E10F | TGACCTTGAATGTGAAGCAC | 58 | 432 |
|  | *PLOD2*-E10R | GGACTCGTAGCCATTAAAAGAA |  |  |
|  | *PLOD2*-E11F | TCCCTGTGAATATCCGTGTT | 58 | 488 |
|  | *PLOD2*-E11R | TCAGGTTGTTTTCTATGACTCC |  |  |
|  | *PLOD2*-E12F | GATGTTGGGAAAGAAGATCC | 58 | 444 |
|  | *PLOD2*-E12R | TTTCAGGATTCCAAGTGGTC |  |  |
|  | *PLOD2*-E13F | AAGGGGCAGTGGTTTATCTC | 58 | 585 |
|  | *PLOD2*-E13R | TCGGTTGCCTATGAATTAGC |  |  |
|  | *PLOD2*-E14F | AAAAGAAGGGCTGAAAGAGC | 58 | 403 |
|  | *PLOD2*-E14R | AACACGCAAACACACAGATG |  |  |
|  | *PLOD2*-E15F | TTTCAGTTGAGTGTCAGTGCT | 58 | 568 |
|  | *PLOD2*-E15R | ACAAGCATTCCCTTTTCTCC |  |  |
|  | *PLOD2*-E16-17F | TTCAGAACCAGGCAAAGATT | 58 | 1281 |
|  | *PLOD2*-E16-17R | GCCTTGGAAAAGAGTGACAT |  |  |
|  | *PLOD2*-E18-20F | GGAAAGGAGTCTACTGACCTT | 58 | 1199 |
|  | *PLOD2*-E18-20R | TCTCAGAGGCAACAAAGCAT |  |  |
| *WNT1* | *WNT1*-E1F | CATTGTCTGCGCCCCTAACC | 58 | 627 |
|  | *WNT1*-E1R | CCCTGCCTGGGGTATCTTAG |  |  |
|  | *WNT1*-E2F | AATTAGTGAGCCTGGGAGAG | 58 | 626 |
|  | *WNT1*-E2R | GGGCAGTAGCTCAGAAAAGT |  |  |
|  | *WNT1*-E3F | TAGCCCTAGAGACCAGCTTT | 58 | 569 |
|  | *WNT1*-E3R | CCTCTATTCTCCGTCTTCGT |  |  |
|  | *WNT1*-E4F | GCAGGGTTTCCAAATCTCAG | 58 | 814 |
|  | *WNT1*-E4R | GTAGGAGGAAGTGGGAGAGA |  |  |
| *P3H1* | P3H1-E3-4-F | CCCTTTCCATCCATAACCTG | 58 | 834 |
|  | P3H1-E3-4-R | GGCTTCCTAGGCCAGTTGTT |  |  |
|  | P3H1-E9-10-F | TGTGACGAGGAGGCAATATC | 58 | 636 |
|  | P3H1-E9-10-R | CCTCACGCAACATAACTCAT |  |  |
|  | P3H1-E14-F | CTTAGGGAA GCCATACTGA AG | 58 | 799 |
|  | P3H1-E14-R | GAGACTCTTGTGCAGGTTCG |  |  |
|  | P3H1-E15-F | AGTTCTTGGTTCCCTCCTTG | 58 | 508 |
|  | P3H1-E15-R | TCCACTCCAAGAGCAGTAGC |  |  |
| *SEC24D* | SEC24D-E1-F | GCTTGAGAATTTGCCCAGGA | 58 | 443 |
|  | SEC24D-E1-R | TCTGAGCCTGAGAACTGAAT |  |  |
|  | SEC24D-E6-F | ATGCCAGTTTGTGTGAGTGT | 58 | 475 |
|  | SEC24D-E6-R | CTCCAGACTTCCATACGCAC |  |  |
|  | SEC24D-E7-F | TCTTTGCAGTAGGATTCAGAGT | 58 | 604 |
|  | SEC24D-E7-R | TGTGCATTACTACAATACGCCA |  |  |
|  | SEC24D-E15-16-F | TCTGGTGGACATTGATTAGACT | 58 | 474 |
|  | SEC24D-E15-16-R | CTCCCCACTGATCATTGTCG |  |  |
|  | SEC24D-E17-18-F | CTCCCATATTTCCTGTTGTTCA | 58 | 1297 |
|  | SEC24D-E17-18-R | GTGCCTGCAACATCATAAGC |  |  |
|  | SEC24D-E21-F | GTCAATTATGGGAATGGGTGC | 58 | 350 |
|  | SEC24D-E21-R | AGGGTGTGCATGTTAAGTCT |  |  |
| **Primer sequences for** **quantitative real-time PCR** | | | | |
| *FKBP10* | qE5F | CTGTCCAGCTAGAGACGCTG | 58 | 81 |
|  | qE5R | TGTAGTGGTAGCGCATGAAG |  |  |
|  | qE10F | GGACCCTGAGAAAACCATAGG | 58 | 106 |
|  | qE10R | AGTCAGATGAGGACGAGGAG |  |  |
|  | qE3-4F | CGACACCAGCTACAGTAAGG | 58 | 109 |
|  | qE4R | CCTTCTCTCTCCAGGACACA |  |  |
|  | qE6F | CCGCAACCACACCTACAATA | 58 | 105 |
|  | qE6R | ATGGTAATTCTCCGGCGTTC |  |  |
|  | qE8-9F | GAGAGTGGAGCCCGGGGAGT | 58 | 109 |
|  | qE9R | CCTTGTGCCACACAAACAGG |  |  |
| **Primers for breakpoint analysis** | | | | |
| *FKBP10* | Up-F | CCCACCTCCACCTCATTTTC | 58 |  |
|  | Down-R | GGTAACTTGCCCAAACTCCA |  |  |
| **Primers for minigene assay** | | | | |
| *FKBP10* | Intron4-ECORI-F | GCCGAATTCAGAGTGGGGCTAGTGTCTTG | 58 | 1531 |
|  | Intron7-XbaI-R | CGTCTAGATCATCTCCTCGACATGCTCC |  |  |
| pcDNA3.1(+) plasmid | CMV-F | CGCAAATGGGCGGTAGGCGTG | 58 |  |
|  | BGH-R | TAGAAGGCACAGTCGAGG |  |  |

**Table S2. Clinical features of probands with autosomal recessive osteogenesis imperfecta in Chinese population**

| **Proband number** | **Age at first fracture (year)** | **Fracture sites** | **Times of fractures (*n*)** | **Frequency of fractures (*n*/year)** | **Scoliosis (Y & N)** | **Height *Z*-score** | **Blue sclerae (Y & N)** | **DI  (Y & N)** | **Hearing loss (Y & N)** | **Independent walking (Y & N)** | **ptosis (Y&N)** |
| --- | --- | --- | --- | --- | --- | --- | --- | --- | --- | --- | --- |
| ***WNT1*** | | | | | | | | | | | |
| PUMC-3 (n=2) | 1.00 | 5 | 3 | 0.75 | Y | -3.78 | N | N | N | N | Y |
| PUMC-10 | 0.03 | 2,3,4,5,6 | 20 | 1.82 | Y | -10.66 | N | Y | N | N | Y |
| PUMC-21 | 0.25 | 2,3,5,6 | 10 | 5.71 | Y | -5.3 | N | N | N | N | Y |
| PUMC-71 | 0.08 | 2,3,4,5,6 | 10 | 1.43 | Y | -6.07 | Y | Y | N | N | Y |
| PUMC-128 (n=2) | 1.00 | 5,6 | 10 | 2.00 | N | -2.90 | N | N | N | Y | N |
| PUMC-145 | 0.08 | 3,4,5,6 | 20 | 0.67 | Y | -10.74 | N | Y | N | Y | N |
| PUMC-154 | 0.33 | 3,5 | 10 | 3.33 | N | -4.92 | Y | Y | N | Y | Y |
| PUMC-158 | 5.00 | 5 | 3 | 0.60 | N | -1.71 | Y | N | N | Y | N |
| PUMC-217 | 0.42 | 5,6 | 4 | 4.00 | N | -0.97 | N | N | N | NA | Y |
| PUMC-221 (n=2) | 0.00 | 2,3,5,6 | 30 | 6.00 | Y | -2.53 | N | Y | N | N | Y |
| PUMC-226 | 0.11 | 2,3,5 | 6 | 1.00 | Y | -5.2 | N | N | N | Y | N |
| PUMC-230 | 0.63 | 3,5 | 20 | 5.71 | Y | 1.32 | N | Y | N | N | Y |
| PUMC-245 | 0.13 | 3,5,6 | 5 | 2.50 | Y | -1.06 | N | N | N | N | N |
| PUMC-247 | 0.03 | 3,5 | 10 | 0.29 | Y | -14.90 | N | N | N | N | Y |
| PUMC-258 | 0.08 | 3,4,5,6 | 4 | 4.00 | N | -3.7 | Y | NA | N | NA | N |
| PUMC-274 | 0.00 | 3,4,5 | 6 | 6.00 | Y | -2.77 | N | N | N | N | Y |
| PUMC-277 | 0.17 | 3,4 | 2 | 2.00 | Y | -1.43 | N | NA | NA | NA | N |
| PUMC-281 (n=3) | 1.50 | 5 | 10 | 1.33 | Y | -2.26 | N | Y | N | N | N |
| PUMC-327 | NA | 3,5,6 | 6 | 1.00 | Y | -2.91 | N | Y | N | N | N |
| PUMC-329 | 0.02 | 4,5,6 | 4 | 1.00 | Y | -4.00 | N | N | N | N | N |
| PUMC-471 (n=2) | 0.00 | 2,3,4,5,6 | 36 | 0.72 | Y | -15.89 | N | Y | N | N | N |
| PUMC-490 | 1.00 | 2,5 | 6 | 1.33 | Y | -4.31 | N | N | N | N | N |
| PUMC-491 | 1.75 | 3,4,5,6 | 25 | 2.50 | Y | -6.73 | N | N | N | Y | Y |
| PUMC-554 | 0.00 | 2,3,5 | 12 | 3.00 | Y | -3.00 | N | N | N | N | Y |
| PUMC-555 | 1.00 | 2,3,5,6 | 9 | 1.10 | Y | -2.54 | N | N | N | N | Y |
| PUMC-572 (n=2) | 1.20 | 5,6 | 25 | 1.10 | Y | -8.00 | Y | N | N | N | N |
| PUMC-586 (n=2) | 1.40 | 5 | 5 | 0.50 | N | -5.20 | N | N | N | Y | N |
| PUMC-612 | 0.00 | 2,3,5,6 | 19 | 3.00 | Y | -5.60 | Y | N | N | N | N |
| PUMC-618 | 7.00 | 3,5 | 5 | 0.50 | Y | -0.50 | N | N | N | N | N |
| PUMC-630 | 1.00 | 3,5,6 | 20 | 2.00 | Y | -1.75 | N | N | N | N | Y |
| ***SERPINF1*** | | | | | | | | | | | |
| PUMC-4 | 1.00 | 2,3,5 | 30 | 2.50 | Y | -3.24 | N | Y | N | Y | N |
| PUMC-33 (n=2) | NA | 3,4 | 3 | 1.50 | NA | NA | N | N | N | N | N |
| PUMC-74 | 2.33 | 3,5 | 18 | 1.50 | Y | NA | N | Y | N | N | N |
| PUMC-150 (n=2) | 1.50 | 2,3,4,5,6 | 55 | 5.79 | Y | -7.19 | N | Y | N | N | Y |
| PUMC-255 (n=2) | 1.25 | 2,3,4,5,6 | 100 | 7.69 | Y | -8.25 | N | N | N | N | N |
| PUMC-275 | 0.50 | 5 | 30 | 8.57 | Y | -2.46 | Y | N | N | NA | N |
| PUMC-306 | 4.00 | 3,5 | 7 | 0.30 | Y | -9.97 | N | Y | N | N | N |
| PUMC-331 | 0.02 | 3,4,5,6 | 25 | 4.17 | Y | -2.93 | N | N | N | N | N |
| PUMC-381 | 1.00 | 3,4,5,6 | 110 | 12.22 | Y | -3.32 | N | Y | N | N | N |
| PUMC-348 | 0.67 | 2,5,6 | 26 | 3.47 | N | -0.33 | Y | N | N | N | N |
| PUMC-422 | 0.92 | 2,3,5,6 | 11 | 2.20 | Y | -5.22 | Y | Y | N | N | N |
| PUMC-496 | 0.58 | 3,4,5 | 20 | 1.82 | Y | -2.66 | N | N | N | Y | N |
| PUMC-482 | 1.50 | 3,4,5 | 30 | 3.33 | N | -4.06 | Y | Y | N | N | Y |
| PUMC-495 | 1.25 | 5 | 6 | 1.36 | N | -1.73 | N | Y | N | N | N |
| PUMC-413 | 2.50 | 3,4,5 | 16 | 5.33 | NA | NA | N | N | N | Y | NA |
| PUMC-527 (n=2) | NA | 2,3,4,5,6 | 45 | 2.00 | Y | -9.33 | N | N | N | N | N |
| PUMC-546 | 1.00 | 2,5 | 15 | 0.50 | Y | -8.00 | Y | N | N | N | N |
| PUMC-585 (n=2) | 0.80 | 3,5 | 90 | 15.00 | Y | -1.76 | Y | N | N | N | N |
| PUMC-595 | 0.50 | 3,5 | 5 | 2.00 | Y | -0.60 | Y | N | N | N | N |
| PUMC-607 | 0.75 | 3;5 | 9 | 3.00 | N | -0.73 | Y | Y | N | N | N |
| PUMC-611 | 0.75 | 5 | 13 | 5.00 | N | 0.54 | Y | N | N | N | N |
| PUMC-624 | 1.20 | 5 | 13 | 3.00 | N | -0.69 | Y | N | N | N | N |
| ***FKBP10*** | | | | | | | | | | | |
| PUMC-68 (n=2) | 0.11 | 2,5 | 6 | 0.46 | Y | -8.90 | N | N | N | Y | N |
| PUMC-121 | 0.83 | 3,5 | 3 | 0.86 | N | 0.17 | N | Y | N | N | N |
| PUMC-157 | 0.25 | 3,5 | 25 | 0.78 | Y | -11.94 | N | Y | N | N | N |
| PUMC-207 (n=2) | 0.06 | 3,4,5,6 | 5 | 0.63 | Y | -5.25 | N | Y | N | N | N |
| PUMC-405 | 0.25 | 2,5,6 | 5 | 2.99 | N | -3.75 | N | Y | N | N | N |
| PUMC-431 | 0.50 | 3,4,5,6 | 21 | 2.33 | N | -2.59 | Y | N | N | N | N |
| PUMC-525 | 0.11 | 2,4,5 | 4 | 6.90 | N | 0.50 | N | NA | N | N | N |
| PUMC-536 | 0.08 | 5,6 | 16 | 2.29 | N | -3.72 | N | Y | N | Y | N |
| PUMC-605(n=2) | 1.20 | 5 | 5 | 2.00 | Y | -2.97 | N | Y | N | N | N |
| PUMC-606 | 0.10 | 4,5,6 | 22 | 2.00 | N | -1.40 | N | N | N | N | N |
| ***SERPINH1*** | | | | | | | | | | | |
| PUMC-285 | 3.00 | 5 | 16 | 1.45 | N | -2.35 | Y | N | N | Y | N |
| PUMC-324 (n=2) | 0.00 | 3,4,5,6 | 10 | 0.67 | Y | -11.61 | N | Y | N | N | N |
| ***CRTAP*** | | | | | | | | | | | |
| PUMC-118 (n=2) | 0.00 | 3,5 | NA | NA | NA | NA | NA | NA | NA | NA | NA |
| PUMC-456 (n=2) | 0.33 | 5 | 2 | 1.00 | N | 0.86 | Y | N | N | Y | N |
| PUMC-582 | 0.20 | 3,5 | 13 | 0.50 | Y | -0.12 | N | N | N | Y | N |
| ***PLOD2*** | | | | | | | | | | | |
| PUMC-320 | 0.08 | 2,3,4,5,6 | 20 | 5.00 | N | -1.09 | N | Y | N | N | N |
| ***SEC24D*** | | | | | | | | | | | |
| PUMC-266 (n=2) | 0 | NA | NA | NA | NA | NA | NA | NA | NA | NA | NA |
| PUMC-204 | 0.33 | 5 | 6 | 1.1 | N | 0.31 | Y | Y | N | Y | NA |
| PUMC-514 | 3 | 3,4,5,6 | 37 | 1.5 | Y | -7.01 | Y | Y | N | Y | N |
| ***P3H1*** | | | | | | | | | | | |
| PUMC-566 | 0 | 2,3,5 | 16 | 4 | N | -2.15 | Y | N | N | Y | N |
| PUMC-590 | 0 | 3;5 | 4 | 2 | N | -1.06 | Y | N | N | N | N |
| PUMC-597 | 0 | 2,3,4,5,6 | 136 | 4 | Y | -6.5 | N | N | N | N | N |

*Note: For each proband, n=1 unless stated on the table, n=number of patients. In column fracture sites, 1=skull, 2=bones of trunk, 3=humerus, 4=ulna and radius, 5=femur, 6=tibia and fibula. Abbreviation: DI=dentinogenesis imperfecta; Y=Yes; N=No; NA=Not Available.

**Table S3. Phenotypic summary of homozygous loss of function in** ***WNT1, SERPINF1, FKBP10, SERPINH1, CRTAP, PLOD2, P3H1* and *SEC24D* in Chinese population**

| **Gene** | **Proband number** | **Number of patients (*n*)** | **Times of fractures (*n*)** | **Frequency of fractures (*n*/year)** | **Scoliosis (yes) percentage** | **Height (cm)** | **Height *Z*-score** | **Blue sclerae (yes) percentage** | **DI (yes) percentage** | **Disability of independent walking (yes) percentage** | **Ptosis (yes) percentage** |
| --- | --- | --- | --- | --- | --- | --- | --- | --- | --- | --- | --- |
| ***WNT1*** | 30 | 38 | 11.83 ± 1.633 ^a^ | 2.230 ± 0.3224 ^a^ | 80.00% ^a^ | 97.40 ± 3.889 ^a^ | -4.667 ± 0.7272 | 20.00% | 32.14% | 22.22% | 46.67% ^a^ |
| ***SERPINF1*** | 22 | 28 | 30.77 ± 6.626 ^b^ | 4.193 ± 0.7986 ^b^ | 70.00% | 108.7 ± 3.154 ^b^ | -3.759 ± 0.7127 | 45.45% | 36.36% | 14.29% | 9.52% ^b^ |
| ***FKBP10*** | 10 | 13 | 11.20 ± 2.764 | 2.124 ± 0.5969 | 40.00% ^b^ | 100.1 ± 6.376 | -3.985 ± 1.230 | 10.00% | 66.67% | 20.00% | 0.00% |
| ***p* value** |  |  | 0.0026 | 0.0149 | 0.0162 | 0.0460 | 0.3978 | 0.0507 | 0.7604 | 0.4951 | 0.0042 |
| ***SERPINH1*** | 2 | 3 |  |  | 50% |  |  | 50% | 50% | 50% | 0.00% |
| ***CRTAP*** | 3 | 5 |  |  | 50% |  |  | 50% | 0.00% | 100% | 0.00% |
| ***PLOD2*** | 1 | 1 |  |  | 0.00% |  |  | 0.00% | 100% | 0.00% | 0.00% |
| ***P3H1*** | 3 | 3 |  |  | 33.33% |  |  | 66.67% | 0.00% | 33.33% | — |
| ***SEC24D*** | 3 | 4 |  |  | 50% |  |  | 100% | 100% | 100% | — |

* Data was shown as mean ± SD, statistical analysis was performed between *WNT1*, *SERPINF1* and *FKBP10* because of the low number involved in genes *SERPINH1*, *CRTAP*, *PLOD2*, *P3H1*, *SEC24D*. p value was calculated for groups *WNT1*, *SERPINF1* and *FKBP10*, and p<0.05 was considered as significant difference. 'a' and 'b' represent statistically significant differences between the two groups.

Figure S1


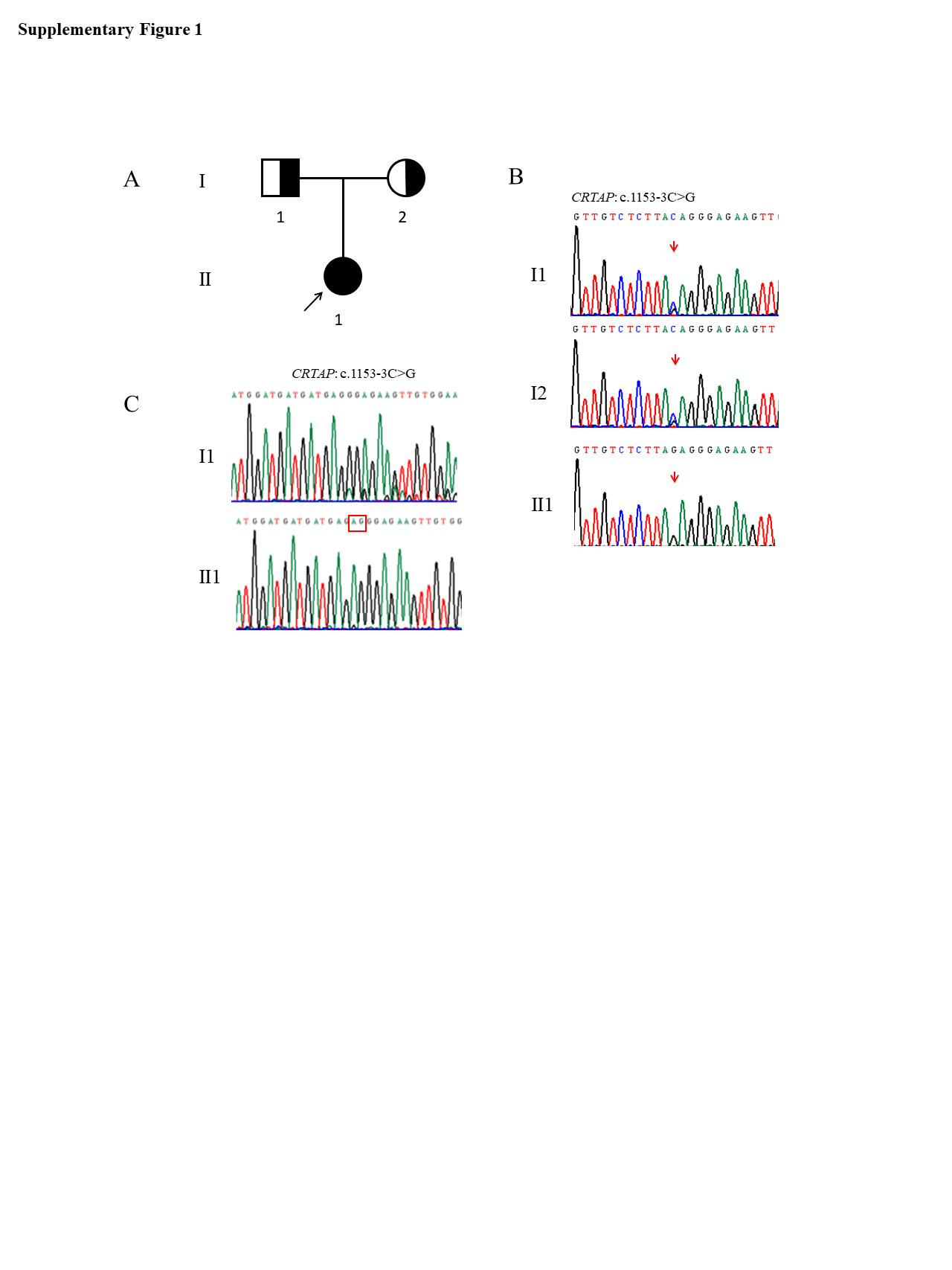


**Fig. S1 Dermal fibroblast analysis confirmed the sequencing result of intronic variant c.1153-3C>G in *CRTAP*.** (**A**) Pedigree information was shown in the family tree. (**B**) Results from genomic DNA sequencing showed an intronic mutation of c.1153-3C>G in *CRTAP* in the proband II1. Parents of the proband were heterozygous carrier of the same variant. (**C**) Sequencing of dermal fibroblasts of I1 and II1 confirmed an insertion of AG in the mutant transcript.

Figure S2


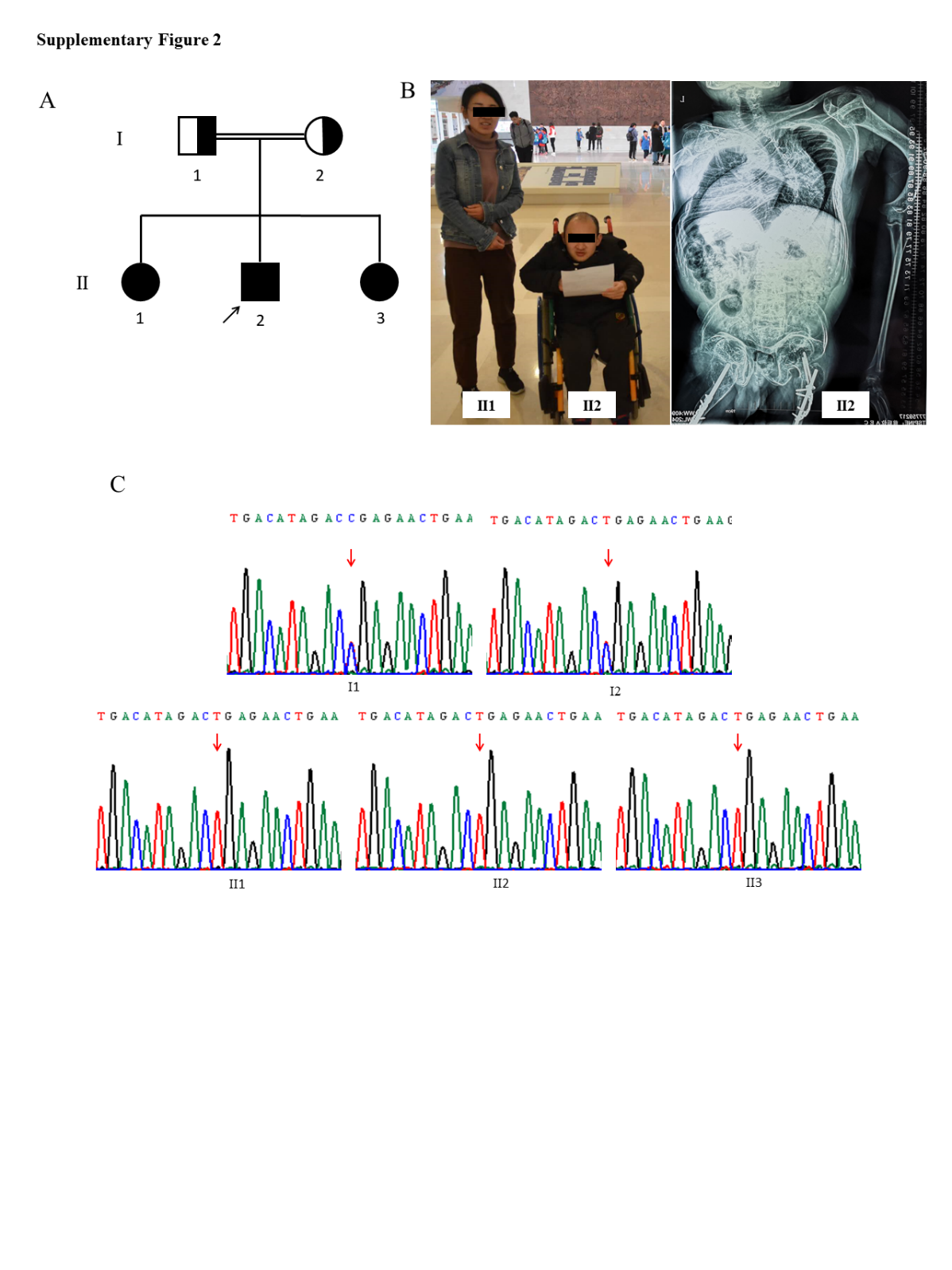


**Fig. S2 A consanguineous family that had the same variant but presented variable expressivity.** (**A**) The pedigree of consanguineous family PUMC-527. (**B**) A photo of patients II1 (mild) and II2 (severe) with a wide difference in their phenotypes. (**C**) Genotype of all the familial members revealed by DNA sequencing.

Figure S3


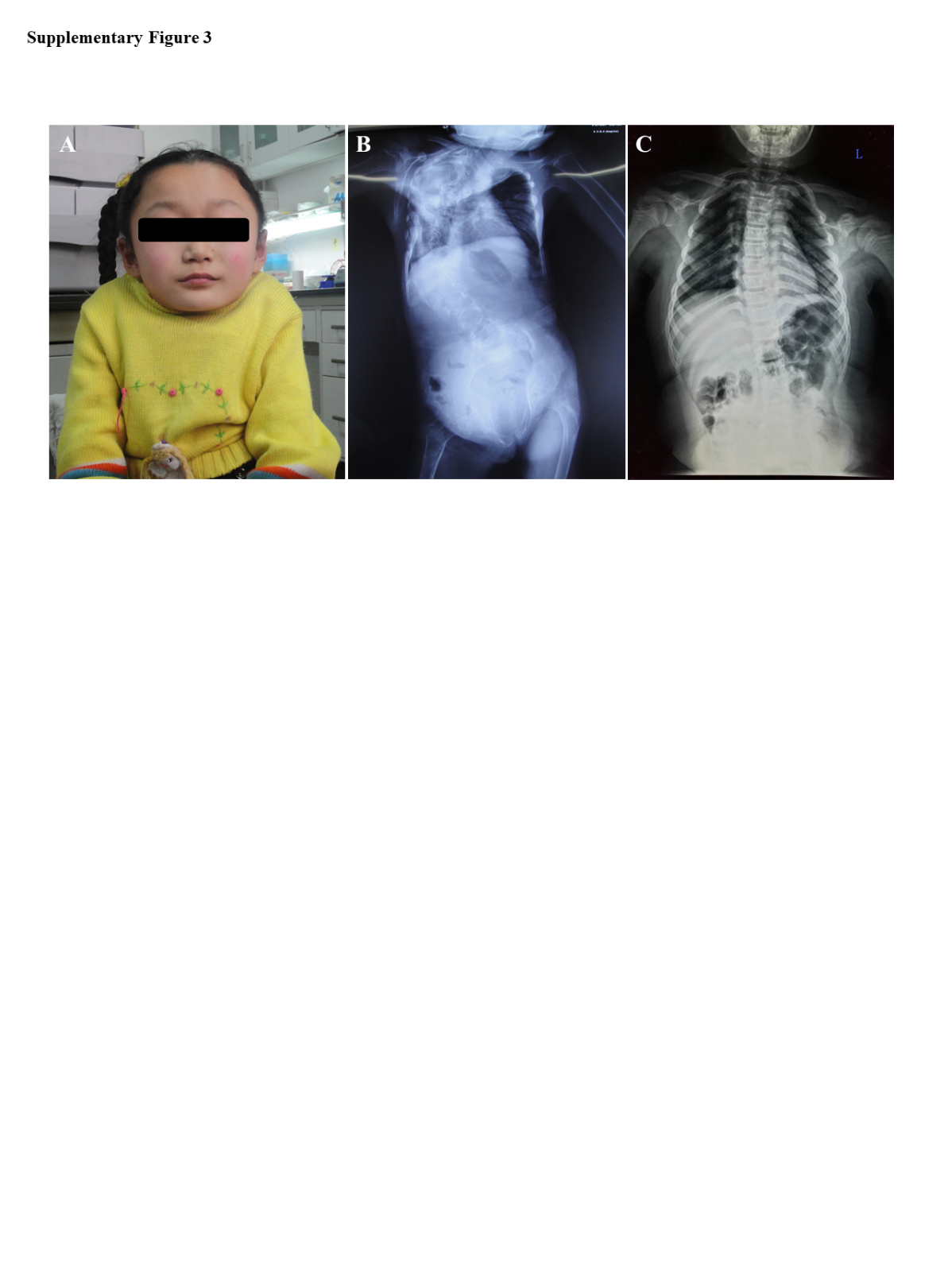


**Fig. S3 Recessive OI patients with variants in *FKBP10* exhibited a unique short neck phenotype. (A-C)** Photo records and X-ray examinations of three individuals with *FKBP10* variants. (A) short neck (PUMC-68), (B) short neck and severe deformity of spinal (PUMC-68), (C) compressible vertebrae (PUMC-121).

Figure S4


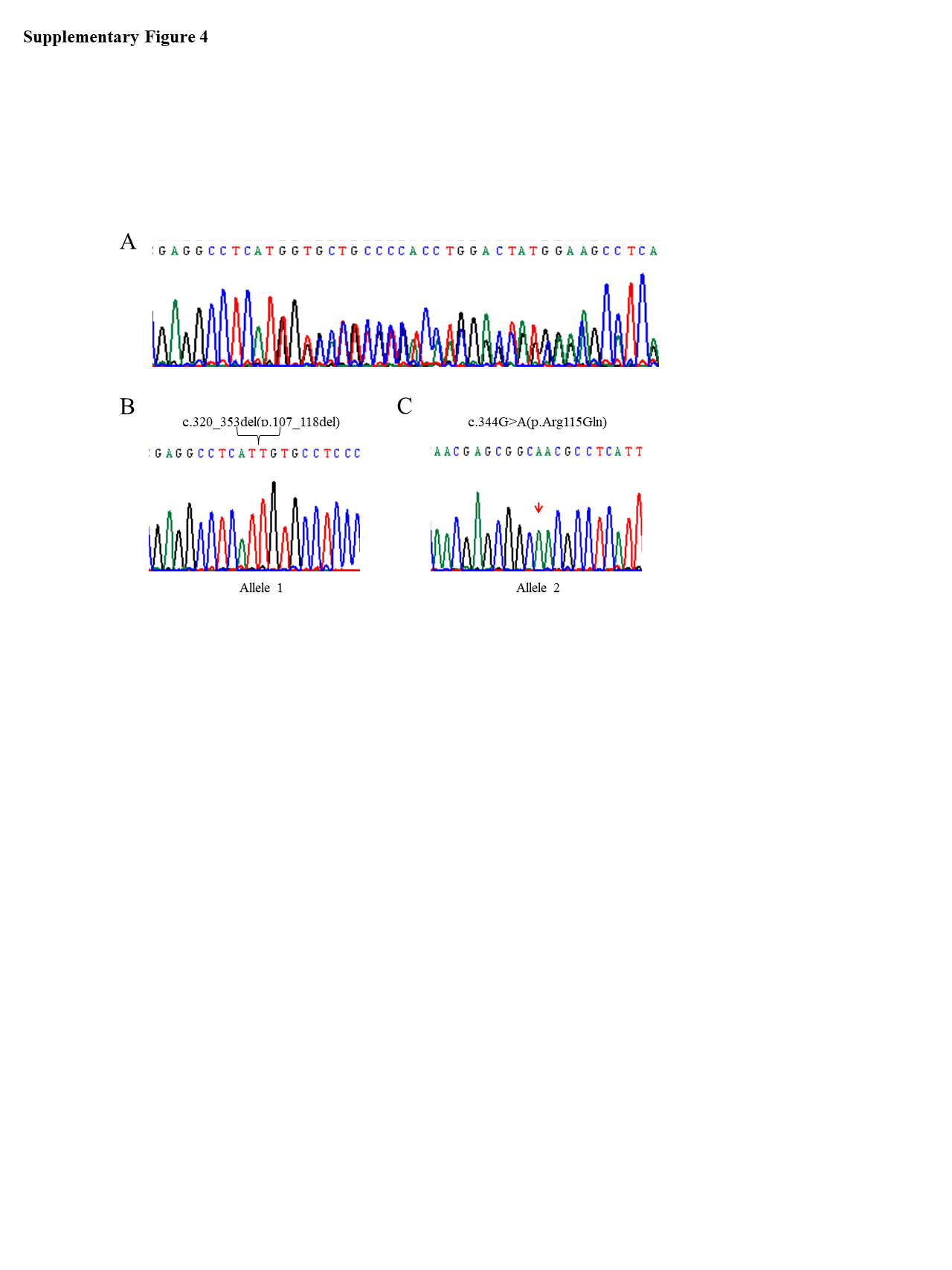


**Fig. S4 A case of micro-deletion and missense mutation in the same region in *FKBP10*.** (**A**) Sanger DNA sequencing of proband PUMC-405 showed disrupted signal in exon 2 in *FKBP10*. (**B-C**) Two mutations was separated after T-clone sequencing: c.320_353del(p.107_118del) (B) and c.344G>A(p.Arg115Gln) which was located within the deletion region (C).

Figure S5


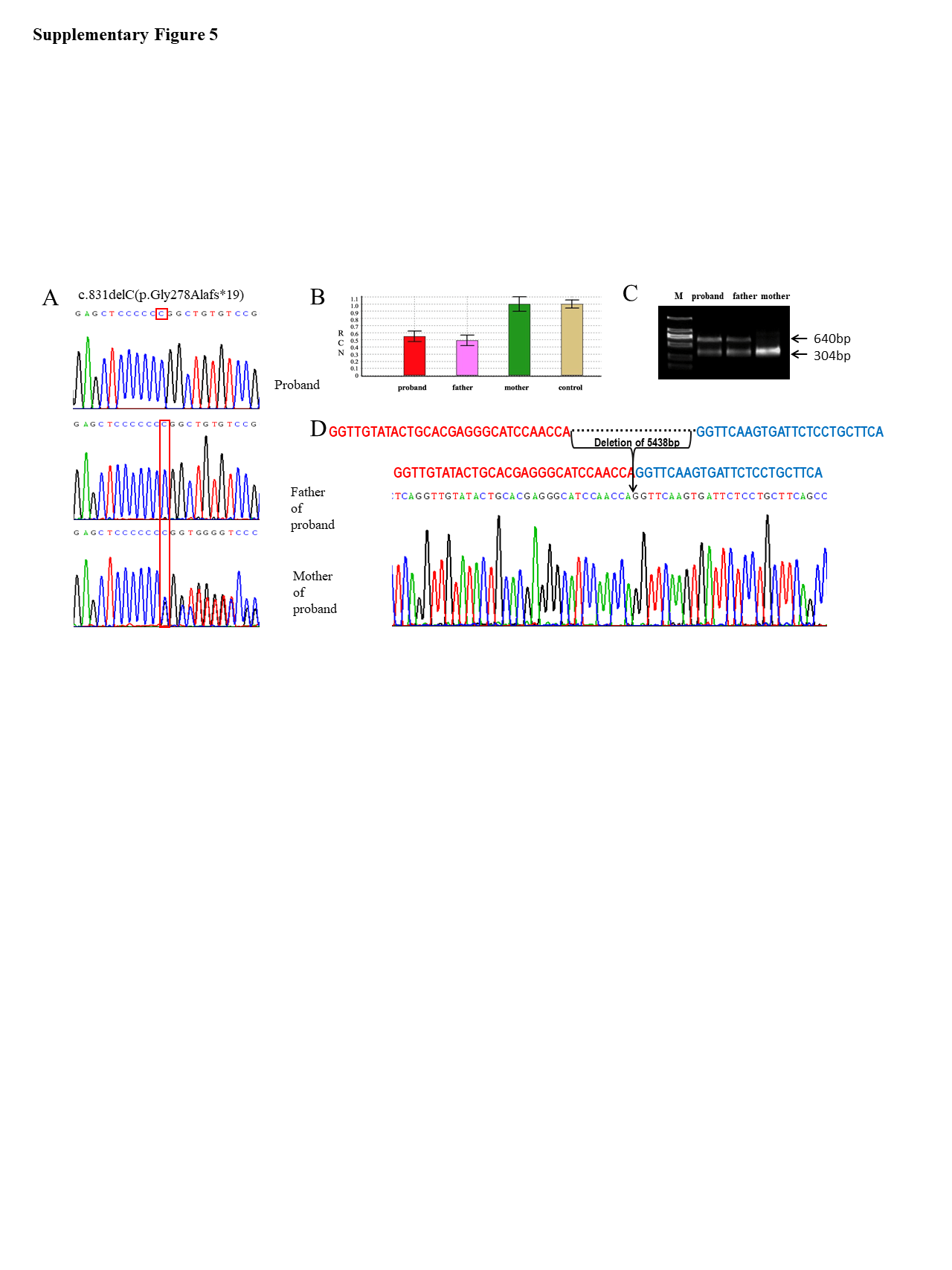


**Fig. S5 A case of gross deletion in *FKBP10*.** (**A**) Sanger sequencing results of the proband and his parents. (**B**) An intragenic deletion was found in the proband and his father indicated by quantitative real-time PCR. The values presented as triplicate determinations ± SD. RCN: Relative Copy Number. (**C**) Breakpoint analysis showed that breakpoint was found in proband and his father (640bp), while absence in the mother of proband because of the existence of original fragment. M: Marker, 640bp: fragment length of the deletion junction product, 304bp: control. (**D**) Sequence chromatograms of the g.41818632_41824069del breakpoint.

Figure S6


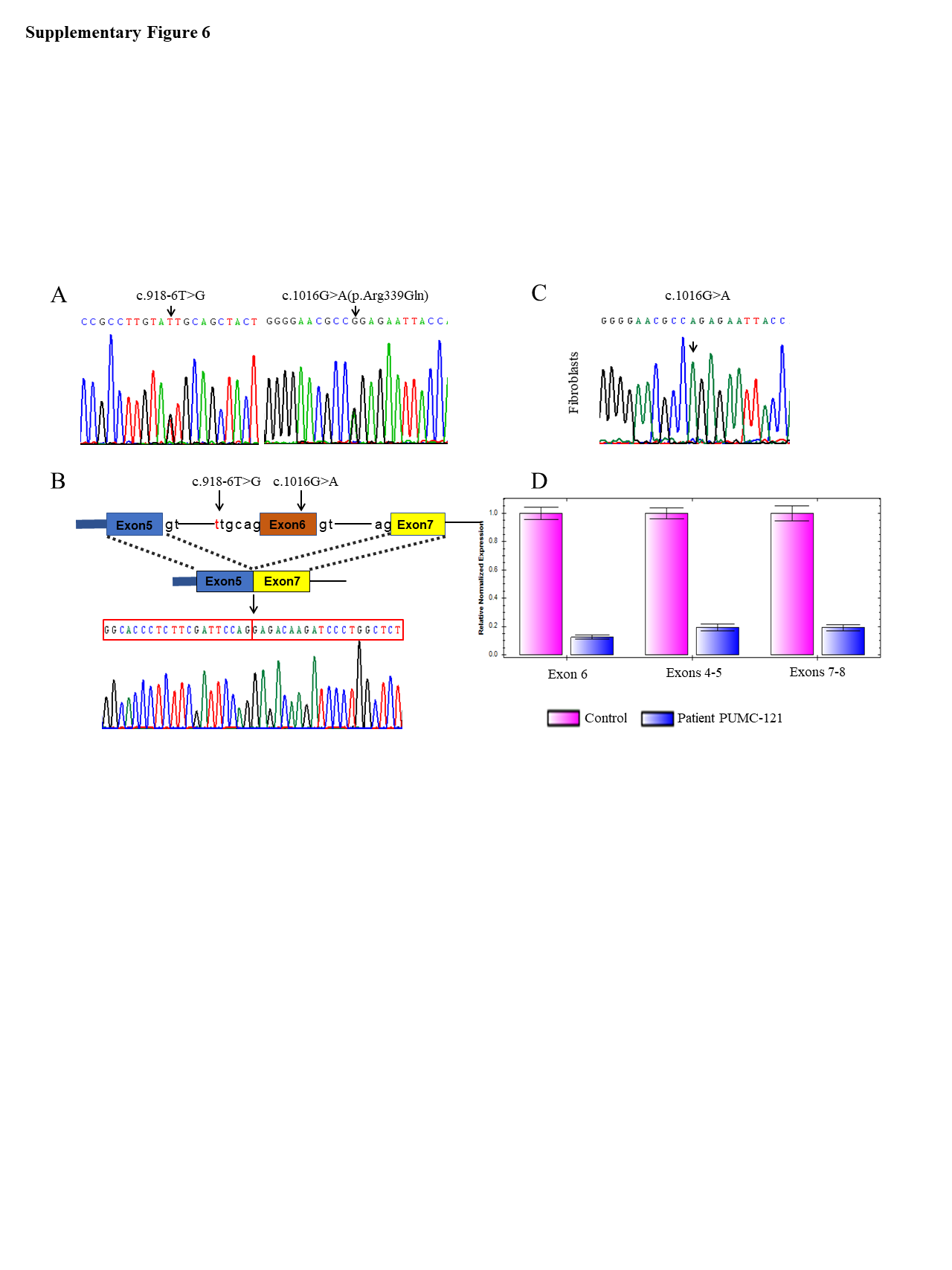


**Fig. S6 Identification of the splicing effect of compound mutations in *FKBP10*.** (**A**) Sanger sequencing indicated a missense variant c.1016G>A(p.Arg339Gln) in exon 6 and an intronic variants c.918-6T>G in adjacent intron 5 in *FKBP10* in patient PUMC-121. (**B**) Minigene assay showed that both c.918-6T>G and c.1016G>A led in skipping of exon 6. (**C**) Sequencing result of RT-PCR product from RNA extracted from the patient’s dermal fibroblasts. (**D**) Relative normalized expression of exon 6, exons 4-5 and exons 7-8 comparing the patient and control. Gene expression in normal control was normalized as 1.
